# Adaptive Smoking Cessation Using Precessation Varenicline or Nicotine Patch: A Randomized Clinical Trial

**DOI:** 10.1001/jamanetworkopen.2023.32214

**Published:** 2023-09-08

**Authors:** James M. Davis, Luisa Masclans, Jed E. Rose

**Affiliations:** 1 Duke Center for Smoking Cessation, Duke University School of Medicine, Durham, North Carolina; 2 Duke Cancer Institute, Durham, North Carolina; 3 Department of Medicine, Duke University School of Medicine, Durham, North Carolina; 4 Duke School of Medicine, Durham, North Carolina

## Abstract

**Importance:**

Adaptive pharmacotherapy, ie, starting a medication regimen and then modifying that regimen based on patient response, is common in many medical domains but is not common in smoking cessation. Recently, studies have found that adaptive treatment using precessation nicotine patches is efficacious for smoking cessation; however, adaptive treatment using precessation varenicline and adaptive treatment in clinical practice settings have not been fully assessed.

**Objective:**

To determine whether adaptive pharmacotherapy leads to higher smoking abstinence rates than standard pharmacotherapy in a clinical practice setting.

**Design, Setting, and Participants:**

This double-blinded stratified placebo-controlled randomized clinical trial compared adaptive treatment with standard treatment for smoking cessation. The study was conducted at a university health system in Durham, North Carolina, from February 2018 to May 2020 and was stopped early due to COVID-19. Data were analyzed as intent-to-treat from May 24, 2021, to February 27, 2022.

**Interventions:**

Participants were allowed to choose varenicline or nicotine patches and were then randomized to adaptive or nonadaptive (standard) treatment. Participants started on their chosen medication (adaptive) or placebo (standard) 4 weeks before their target quit day. Two weeks later, participants were assessed for treatment response. Adaptive participants who did not decrease daily cigarettes smoked by at least 50% (nonresponders) received bupropion in addition to their chosen medication. Participants in the adaptative treatment group who did decrease daily cigarettes smoked by at least 50% (responders) and participants in the standard treatment group received additional placebo bupropion. Participants in the standard treatment group received varenicline starting 1 week before the target quit date or nicotine patches starting on the target quit day. All participants received brief behavioral support.

**Main Outcome and Measures:**

The main outcome was biochemically verified 30-day continuous smoking abstinence 12 weeks after their target quit smoking day. Other measures included demographic characteristics, smoking history, and repeated smoking assessments.

**Results:**

Of the planned 300 participants, a total of 188 participants (mean [SD] age, 49.1 [12.5] years; 102 [54%] female) were enrolled before the trial was stopped because of the COVID-19 pandemic. A total of 127 participants chose to use varenicline, including 64 randomized to adaptive treatment and 63 randomized to standard treatment, and 61 participants chose to use nicotine patches, including 31 randomized to adaptive treatment and 30 randomized to standard treatment. At baseline, participants smoked a mean (SD) of 15.4 (7.3) cigarettes per day. At 12 weeks after the target quit day, biochemically verified 30-day continuous smoking abstinence was observed in 23 of 95 participants (24%) in the adaptive treatment group and 8 of 93 participants (9%) in the standard treatment (odds ratio [OR], 3.38; 95% CI, 1.43-7.99; *P* = .004); among participants who used varenicline, 30-day continuous abstinence was 18 participants (28%) in the adaptive treatment group, and 5 participants (8%) in the standard treatment group (OR, 4.54; 95% CI, 1.57-13.15); among participants who used nicotine patches, 30-day continuous abstinence was 5 participants (16%) in the adaptive treatment group and 3 participants (10%) in the standard treatment group (OR, 1.73; 95% CI, 0.38-7.99). Sleep problems were more common for participants in the varenicline adaptive treatment group than in the varenicline standard treatment group (rate ratio, 1.74; 95% CI, 1.18-2.58; *P* = .03).

**Conclusions and Relevance:**

This randomized clinical trial found that adaptive pharmacotherapy was efficacious for smoking cessation treatment in a practice setting.

**Trial Registration:**

ClinicalTrials.gov Identifier: NCT02501265

## Key Points

**Question:**

Is adaptive pharmacotherapy effective for smoking cessation compared with nonadaptive standard pharmacotherapy?

**Findings:**

In this randomized clinical trial, 188 smokers chose between varenicline and nicotine patches and were then randomized to adaptive or standard treatment. Biochemically verified 30-day continuous smoking abstinence at 12 weeks after the target quit date was significantly higher for the adaptive treatment group than the standard treatment group.

**Meaning:**

These findings suggest that adaptive pharmacotherapy was effective for smoking cessation.

## Teaser

This randomized clinical trial assesses the efficacy of adaptive pharmacotherapy compared with standard pharmacotherapy using varenicline or nicotine patches for smoking cessation.

## Introduction

Adaptive treatment, ie, assessing the patient’s response to an initial medication and then modifying the medication regimen based on the patient’s response, is a common medical practice.^1,2^ However, this approach has not been widely adopted for smoking cessation. The US Food and Drug Administration (FDA) has approved specific protocols for smoking cessation medications in which medications are started on the quit-smoking day (nicotine patch, gum, lozenge inhaler nasal spray) or 1 week before the quit-smoking day (bupropion, varenicline).^3^ Research has demonstrated acceptable safety and efficacy of starting medications 4 weeks before the quit smoking day for varenicline,^4,5,6^ bupropion,^7^ and nicotine patches^8,9^ and opened the door to the exploration of precessation adaptive treatment. It may be helpful to conceptualize precessation adaptive smoking cessation pharmacotherapy as having 3 components: extended precessation pharmacotherapy, assessment of response to precessation pharmacotherapy, and adaptation of medication regimens for nonresponders.

Regarding extended precessation pharmacotherapy, trials have now shown that 4, 6, and 12 weeks of precessation varenicline all show higher smoking abstinence rates than 1 week of precessation varenicline.^4,5,6^ The efficacy of precessation nicotine patches is less certain, with the most recent meta-analysis (8 trials, 2813 participants) showing no significant association of precessation nicotine patch use with smoking abstinence (RR, 1.16, 95% CI: 0.97-1.38).^8^

Regarding assessment of response to precessation pharmacotherapy, studies have shown that precessation smoking reduction was associated with postcessation smoking abstinence when smokers used precessation varenicline or nicotine patches.^4,10,11^ A study on precessation nicotine patches found that responders (ie, those who showed ≥50% precessation carbon monoxide [co] reduction) showed a 2-fold higher smoking abstinence rate than nonresponders.^12^ A study on precessation varenicline found that responders showed a 2.95-fold higher smoking abstinence rate than non-responders.^4^

Regarding adapting of medication regimens for nonresponders, a 2013 study^13^ found that precessation nicotine patch nonresponders could be rescued by adding bupropion 150 mg twice daily, resulting in significantly higher 6-month smoking abstinence compared with participants who continued using nicotine patch alone. To our knowledge, there have been no studies assessing adaptive treatment in clinical populations with minimal exclusion criteria, nor studies assessing adaptive treatment for smokers using precessation varenicline.

## Methods

### Design, Funding, and Registration

This phase 2 double-blind placebo-controlled stratified randomized clinical trial was approved by the Duke University Health System Institutional Review Board, and all participants provided written informed consent prior to enrollment. The trial was designed to compare biochemically verified 12-week smoking abstinence in daily smokers randomized to adaptive or standard pharmacotherapy in a clinical practice setting. The trial protocol and statistical plan are provided in Supplement 1.

### Participants

The study was conducted at Duke University Health System, a large university health system in central North Carolina. Potential participants were daily smokers who had been referred to a clinical smoking cessation program. Potential participants were offered free medications and compensation for study participation. Inclusion criteria were that participants must be daily smokers for at least 1 year, aged at least 18 years, and willing to attempt smoking cessation. Exclusion criteria included pregnancy, use of multiple tobacco products, current use of smoking cessation medications, allergy or intolerance to study medications, medical illness requiring hospitalization, psychiatric illness requiring hospitalization, alcohol or sedative dependence requiring treatment, significant recent illicit substance use, or baseline exhaled co less than 7 ppm. Participant medical history was assessed through a medical evaluation, surveys and the electronic health record. Standardized questionnaires used included the Fagerström Test for Cigarette Dependence,^12^ Drug Abuse Screening Test,^14^ Alcohol Use Disorders Identification Test,^15^ Patient Health Questionnaire-Depression–9-item,^16^ and the Generalized Anxiety Disorder–7-item.^17^ Full inclusion and exclusion criteria are available in the study protocol in Supplement 1.

### Stratification, Randomization, and Blinding

After enrollment, participants were stratified by medication choice. Using FDA information, participants were asked to choose between using nicotine patches or varenicline. After stratification, participants in the varenicline and nicotine patch groups were randomized 1:1 to adaptive treatment or standard treatment groups. Randomization used a predetermined computer-generated random sequence within each stratified group to ensure an equal number of adaptive and standard participants among those who chose varenicline and those who chose nicotine patches. Participants and staff were blinded to group allocation. During study operations, only the research pharmacy staff was aware of group allocation, and they dispensed placebos with visually identical packaging for both medications.

### Interventions

#### Behavioral Treatment

At the baseline visit, all participants were provided with 20 minutes of evidence-based smoking cessation counseling by a tobacco treatment specialist. Smoking cessation counseling including health education, motivational support, and instructions on medication use.

#### Medication Treatment

Adaptive treatment participants were provided with their chosen medication (nicotine patch or varenicline) 4 weeks before the target quit day. Varenicline was up-titrated over the first week of treatment to 1 mg twice daily. The nicotine patch was started at 14 to 21 mg based on baseline cigarettes smoked per day. Standard treatment participants who chose to use varenicline were provided with a placebo during starting at 4 weeks before the target quit day, then received varenicline starting 1 week before the target quit day. Standard treatment participants who chose to use nicotine patches received placebo patches starting 4 weeks before the target quit day, then received real nicotine patches starting on the target quit day. All medications were continued for 12 weeks after the target quit day. Similar to a previous study on adaptive treatment that directed participants to continue ad libitum smoking during precessation treatment,^18^ participants in the study were directed to continue smoking as usual during precessation treatment so that precessation smoking reduction would more likely reflect a treatment response to medication rather than willful smoking reduction. Two weeks after starting medications, participants attended a medication-response visit. At this visit, adaptive treatment participants who did not decrease smoking by at least 50% were classified as nonresponders, and bupropion 150 mg twice daily was added to their precessation regimen. Adaptive treatment participants who decreased cigarettes smoked per day by at least 50% were classified as treatment responders, and placebo bupropion was added to their regimen. Standard treatment participants were assessed at the medication-response visit and provided with placebo bupropion.

### Outcome Measures

Study participants were asked to attend 4 study assessment visits: the baseline visit, medication response visit (2 weeks before the target quit day), and 2 post–quit day visits (2 and 12 weeks after the target quit day). Participants were paid $190 if they completed all 4 study visits. Baseline variables included age, sex, race, ethnicity, smoking history, nicotine dependence, and psychiatric diagnoses. Race and ethnicity were determined via self-report. Race was classified as American Indian or Alaska Native, Asian, Black or African American, Native Hawaiian or Other Pacific Islander, White, more than 1 race, and unknown, and ethnicity was classified as Hispanic or Latino or not Hispanic or Latino. Race and ethnicity were included in the analysis as potential covariates of our primary outcome.

The primary outcome was 30-day continuous smoking abstinence 12 weeks after the target quit day, verified by expired co breath test of less than 7 ppm (ppm). We used 12-week postquit outcomes instead of 6- or 12-month outcomes in this study because the study used a clinical population with modest compensation, which may have resulted in higher attrition at later timepoints. Additionally, comparative abstinence rates between treatments at 12 weeks have been associated with outcomes at 6 and 12 months.^19,20,21^ Secondary outcome measures included self-reported cigarettes smoked per day and expired co breath testing at each study visit. Adverse events were assessed at each assessment visit using open-ended questions and direct inquiry. For direct inquiry, participants were asked about the presence and intensity (range, 1-7; higher score indicating more intensity) of side effects commonly reported for medications used in this study including: nausea, vomiting, insomnia, vivid dreams, agitation, anxiety, depression, headache, and rash. A study physician determined the likelihood that moderate and severe adverse events might be study related.

### Sample Size

The sample size was based on a 2-tail significance test with 80% power and a 15% attrition rate (from our previous studies at our center). We referenced smoking abstinence rates from available trials that did not enroll a clinical population, and showed an adaptive treatment rate of 39.7%, and standard treatment of 23.4% and an absolute difference between groups of 16.3 percentage points. The study was initially designed to have sample size of 300 participants but was stopped early due to COVID-19 pandemic–related restrictions on in-person visits. After restrictions were lifted, funding was no longer available for the study, and analyses were conducted on the enrolled sample.

### Statistical Analysis

The primary outcome for the study was 30-day continuous smoking abstinence 12 weeks after the target quit day, verified by expired co breath test result of less 7 ppm. Participants with missing smoking abstinence data were categorized as nonabstinent. All analyses of smoking abstinence were intent-to-treat. The primary analysis compared all adaptive treatment participants with all standard treatment participants using the Score Test from a stratified logistic regression model, equivalent to the Cochran-Mantel-Haenszel method. The logistic regression included a single factor (adaptive vs standard treatment), with likelihood maximized in 2 strata (nicotine patch and varenicline) and results combined to estimate a common OR across the nicotine patch and varenicline randomization strata.^20^ Statistical tests were 2-sided using α = .05 and 2-sided 95% CIs. Abstinence outcomes were not adjusted based on baseline covariates because covariates did not differ between groups. The study was not powered to detect an interaction between assigned treatment and medication choice (eg, adaptive vs standard treatment among participants who chose varenicline); thus, these results are reported descriptively. Adaptive and standard treatment co levels were compared post hoc over time across all assessment visits using a random intercept regression model with group-by-time interaction term. All analyses were performed using SAS statistical software version 9.4 (SAS Institute), with March 30, 2021, updates. Data were analyzed from May 24, 2021, to February 27, 2022.

## Results

### Participants

**Figure 1.**
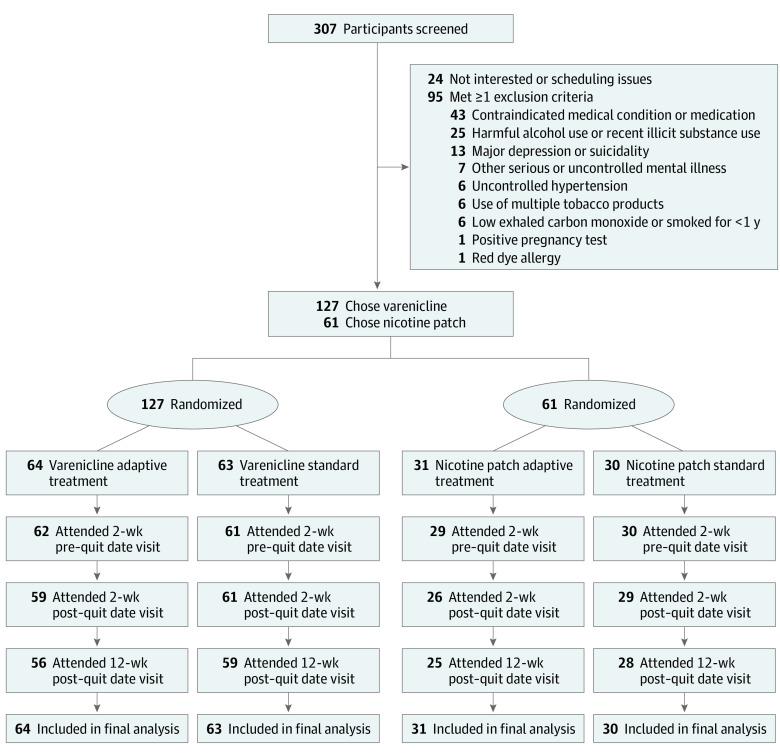
Participant Recruitment Flowchart All enrolled participants were included in intention-to-treat analyses used to determine primary outcome.

The first enrollment was February 15, 2018, and the last study visit was March 11, 2020. The study goal was to enroll 300 participants, but it was stopped early due to the COVID-19 pandemic. Of 307 individuals screened, 24 were not interested in the study, and 95 were excluded based on exclusion criteria. A total of 188 participants (mean [SD] age, 49.1 [12.5] years; 102 [54%] female) were enrolled and randomized, with 127 participants choosing to use varenicline, including 64 randomized to adaptive treatment and 63 randomized to standard treatment, and 61 participants choosing to use nicotine patches, including 31 randomized to adaptive treatment and 30 randomized to standard treatment. At baseline, the mean (SD) Fagerstrom Test for Cigarette Dependence^15^ score was 5.0 (2.1), and participants smoked a mean (SD) of 15.4 (7.3) cigarettes per day. In the full sample, there were no American Indian or Alaska Native participants, 3 Asian participants (2%); 80 Black or African American participants (43%), 1 Native Hawaiian or Other Pacific Islander participant (1%), 98 White participants (52%), and 6 participants (3%) who identified as more than 1 race; 4 participants were Hispanic or Latino (2%) and 184 participants (98%) were not Hispanic or Latino (Table 1). There were 49 participants (31%) with a major psychiatric diagnosis (eg, depression, anxiety, bipolar, schizophrenia) (Table 1). Baseline characteristics were similar between randomized groups on all variables assessed, including age, sex, race, ethnicity, cigarettes per day, nicotine dependence, and incidence of a psychiatric diagnosis. Of 188 randomized participants, 137 attended the 12-week postquit time point visit (primary outcome) and 51 (27%) were lost to follow-up, with similar attrition rates across groups (Figure 1).

**Table 1.** Baseline Variables in the Study Cohort

Variable	Participants, No. (%)				
	Total (N = 188)	Varenicline		Nicotine patch	
		Adaptive (n = 64)	Standard (n = 63)	Adaptive (n = 31)	Standard (n = 30)
Age, mean (SD), y	49.1 (12.5)	49.1 (12.6)	50.4 (10.5)	50.3 (11.6)	45 (16.3)
Cigarettes smoked per d in past 7 d, mean (SD), No.	15.4 (7.3)	16.1 (7.0)	16.4 (7.4)	13.4 (5.8)	14.1 (8.5)
FTND score at screening, mean (SD)	5.0 (2.1)	4.9 (2.1)	5.4 (2.4)	4.8 (1.5)	4.8 (1.7)
Sex					
Female	102 (54)	35 (55)	30 (48)	19 (61)	17 (57)
Male	86 (46)	28 (44)	33 (52)	12 (39)	13 (43)
Race					
American Indian or Alaska Native	0	0	0	0	0
Asian	3 (2)	1 (2)	1 (2)	0	1 (3)
Black or African American	80 (43)	19 (30)	23 (37)	17 (55)	21 (70)
Native Hawaiian or Other Pacific Islander	1 (1)	1 (2)	0	0	0
White	98 (52)	41 (64)	38 (60)	13 (42)	6 (20)
≥1	6 (3)	2 (3)	1 (2)	1 (3)	2 (7)
Unknown or not reported	0	0	0	0	0
Ethnicity					
Hispanic or Latino	4 (2)	1 (2)	1 (2)	2 (7)	0
Not Hispanic or Latino	184 (98)	63 (98)	62 (98)	29 (94)	30 (100)
Diagnosis of psychiatric illness	49 (31)	17 (32)	17(32)	8 (30)	7 (28)

Abbreviation: FTND, Fagerstrom Nicotine Dependence Test.

### Efficacy

**Figure 2.**
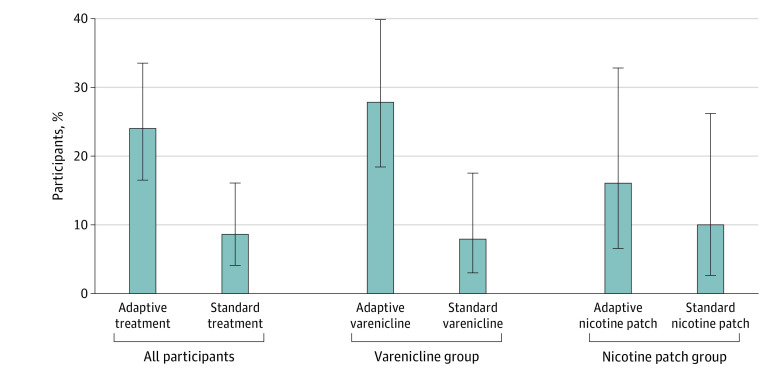
Biochemically Verified 30-Day Continuous 12-Week Postquit Smoking Abstinence All participants: odds ratio, 3.38 (95% CI, 1.43-7.99); *P* = .004. Precessation varenicline: odds ratio, 4.54 (95% CI, 1.57-13.15). Precessation nicotine patch: OR, 1.73 (95% CI, 0.39-7.99). Error bars indicate 95% CIs.

Among all participants, intent-to-treat 12-week postquit biochemically verified 30-day continuous smoking abstinence was 23 of 95 participants (24%) in the adaptive group and 8 of 93 participants (9%) in the standard treatment group (OR, 3.39; 95% CI, 1.43-7.99; *P* = .004). Among participants using varenicline, smoking abstinence was 18 participants (28%) in the adaptive treatment group and 5 participants (8%) in the standard treatment group (OR, 4.54; 95% CI, 1.57-13.15). Among participants who chose the nicotine patch, smoking abstinence was confirmed in 5 participants (16%) in the adaptive treatment group and 3 participants (10%) in the standard treatment group (OR, 1.73; 95% CI, 0.39-7.99) (Figure 2). Among all participants in the adaptive treatment group, the 12-week smoking abstinence rate for responders was 6 of 12 responders (50%) and 17 of 48 nonresponders (25%) in the varenicline group and 3 of 13 responders (23%) and 3 of 15 nonresponders (20%) in the nicotine patch group. Statistical comparison was not conducted for these groups because responder status occurred as a postrandomization event. Intent-to-treat biochemically verified 7-day smoking abstinence at the 2-week postquit visit was 32% in the adaptive treatment group and 11% in the standard treatment group (OR, 3.30; 95% CI, 1.49-7.28).

**Figure 3.**
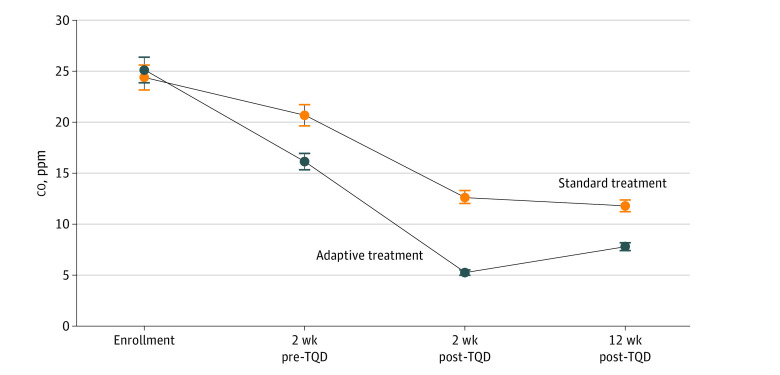
Mean Expired Carbon Monoxide (co) for Adaptive and Standard Treatment Significant group-by-time interaction across all postbaseline time points: *P* = .001. TQD indicates target quit date; error bars, 95% CIs.

Figure 3 shows the change in expired co across all adaptive treatment vs all standard treatment participants. Compared with standard treatment, participants in the adaptive treatment group showed significantly lower co at all postbaseline time points (eg, 12 weeks: 9.41 ppm [38.6% reduction] vs 17.38 ppm [69.0% reduction]) with time-by-group interaction across all time points (*P* = .001).

### Safety

Among all 188 participants, we identified 1 serious adverse event: 1 participant (2%) in the varenicline standard treatment group died. This death was determined to be related to stage 4 cancer and not study-related. There were no other reports of death, life threatening events, hospitalization, persistent or significant disability or incapacity. Among all participants, 9 reported moderate side effects (requiring medical evaluation or change in daily routine) (4 participants in the varenicline adaptive group; 1 participant in the varenicline standard group; 3 participants in the nicotine patch adaptive group; 1 participant in the nicotine patch standard group). When mild adverse events (requiring no medical evaluation or a change in daily routine) were included, 159 participants reported at least 1 adverse event (Table 2). There were no significant differences between the incidence of adverse events between adaptive and standard treatment groups, except for sleep problems, which were more common in varenicline participants randomized to adaptive treatment compared with those randomized to standard treatment (RR, 1.74; 95% CI, 1.18-2.58; *P* = .03).

**Table 2.** All Adverse Events Reported^a^

Group	Participants, No. (%)						
	Any	GI^b^	Psychiatric^c^	Sleep^d^	Neurological^e^	Rash	Other
All participants	159 (85)	100 (53)	44 (23)	88 (47)	57 (30)	25 (13)	67 (36)
Varenicline adaptive	58 (91)	41 (64)	21 (33)	39 (61)	20 (31)	1 (2)	25 (39)
Varenicline standard	49 (78)	32 (51)	10 (16)	22 (35)	17 (27)	4 (6)	15 (24)
Patch adaptive	27 (87)	12 (39)	6 (19)	18 (58)	11 (36)	12 (39)	16 (52)
Patch standard	25 (83)	15 (50)	7 (23)	9 (30)	9 (30)	8 (27)	11 (37)

Abbreviation: GI, gastrointestinal.

^a^ Includes mild and nonstudy related adverse events.

^b^ Includes constipation, dry mouth, nausea, vomiting, and gas.

^c^ Includes anxiety and anger or agitation.

^d^ Includes insomnia and vivid dreams.

^e^ Includes dizziness and headaches.

## Discussion

This randomized clinical trial found that adaptive treatment was more efficacious than standard treatment for daily smokers recruited from a smoking cessation clinic and allowed to choose between varenicline and nicotine patches. Additionally, the results suggest that adaptive treatment with extended (4-week) precessation varenicline may be more effective than standard varenicline treatment starting 1 week before the target quit date. This study contributes to the existing literature, which thus far only supports the use of adaptive treatment using precessation nicotine patches and has not assessed smokers in clinical settings. The trial used a clinical population with minimal exclusion criteria so that findings might be more readily generalizable to smokers seen in a clinical setting. A component of this trial’s design was allowing participants to select their medication. Another important element was to use minimal exclusion criteria, such that only 31% of screened participants were excluded from the trial. This resulted in a study sample with rates of psychiatric comorbidity (30.8%) similar to that in US smokers (35%).^22,23^ Of note, 72.8% of smoking cessation trials exclude some or all potential participants due to mental illness.^24^ The study sample also had a higher portion of Black or African American participants (43%) than is found in the general US population (13.6%) and in the US population of smokers (20.4%).^25,26^

An important perspective on trial outcomes is provided by studies showing that varenicline plus bupropion was more effective than varenicline alone with continuous 12-week postquit smoking abstinence between groups (OR, 1.89; 95% CI, 1.07-3.35; *P* = .03 vs OR, 1.49; 95% CI, 1.05-2.12; *P* = .03).^27,28^ Our study may have found a larger treatment effect between groups because in our study, the varenicline standard treatment arm did not use extended precessation varenicline.

To our knowledge, this is first trial that tested adaptive smoking cessation treatment in a clinical population with minimal exclusion criteria. As such, our best estimates for smoking abstinence in the standard treatment group were derived from randomized trials that used more restrictive eligibility criteria, eg, excluding smokers with physical or psychiatric illness.^4,13^ These studies provided estimates of smoking abstinence rates of 39.7% for adaptive treatment and 23.4% for standard treatment, an absolute difference between groups of 16.3 percentage points and a 1.7-fold increase in efficacy. To put this in perspective, nicotine patches, which are considered to have a meaningful treatment effect, lead to a 1.7-fold increase in efficacy over placebo.^29^ In this trial, the abstinence rate was 24% for adaptive treatment and 9% for standard treatment, with an absolute difference of 15.6 percentage points and a 2.8-fold increase in efficacy. Thus, although abstinence rates for all participants were lower in this trial than in our reference studies, the size of the absolute and relative effects between groups were as large as expected and large enough to be clinically meaningful.

### Limitations

This study has some limitations, including the exclusion of people with symptomatic alcohol dependence or substance use. Additionally, few or no Alaska Native, American Indian, Asian, Hispanic or Latinx, multiracial, or Pacific Islander people enrolled, limiting generalizability to these populations. Additionally, study participants were given free medications and modest compensation for study visits, which are both elements that reduce generalizability. Another limitation emerged from the fact that that the study was stopped early due to COVID-19, resulting in a sample size of 188 instead of 300, with our lower sample size resulting in the relatively wide 95% CI for our main outcome. The relatively high percentage (27%) of participants lost to follow up might be explained by modest compensation and high comorbidity. Participants with missing abstinence data were counted as nonabstinent, which adds to the uncertainty of study outcomes. An additional limitation is that the study was designed to compare adaptive treatment as a whole with standard treatment as a whole and was not designed to assess individual components of adaptive treatment (effects of extended precessation treatment, effects of adding a second medication for nonresponders). Overall, these limitations were seen as decreasing the strength of but not negating our conclusions.

## Conclusions

This randomized clinical trial in daily smokers from a smoking cessation clinic with medication choice and limited exclusion criteria found higher smoking abstinence rates among participants randomized to adaptive compared with those randomized to nonadaptive pharmacotherapy. Specifically, these findings provide support for the use of precessation varenicline and precessation nicotine patches in an adaptive treatment regimen in which bupropion is provided to treatment nonresponders. This study’s design provides greater generalizability to clinical populations. Our findings support an evolving body of literature on adaptive treatment.

## Notes

Supplement 1.
Trial Protocol and Statistical Analysis Plan

Supplement 2.
Data Sharing Statement
