# Supplementary material for: Adaptive Smoking Cessation Using Precessation Varenicline or Nicotine Patch: A Randomized Clinical Trial
Source: JAMA Netw Open. 2023 Sep 8;6(9):e2332214. doi: 10.1001/jamanetworkopen.2023.32214 (PMC10492187; doi:10.1001/jamanetworkopen.2023.32214)
Supplement: Supplement 1. — Trial Protocol and Statistical Analysis Plan [file jamanetwopen-e2332214-s001.pdf]

A Supportive Care-Drug Intervention Study

Randomized Controlled Smoking Cessation Trial on Adaptive Pharmacotherapy

DUKE CANCER INSTITUTE

A National Cancer Institute-designated Comprehensive Cancer Center

Sponsor:	James Davis, MD – Duke Cancer Institute
Funding Source:	National Institutes of Health
Protocol Source:	James Davis, MD - Duke Cancer Institute
Duke IRB#:	Pro00072077

Principal Investigator
James Davis, MD
2424 Erwin Road, Suite 201
Durham, NC
Phone: 919-668-5055
Fax: 919-668-5088
james.m.davis@duke.edu

Co-Investigator
Santanu Datta, Ph.D.
santanu.datta@duke.edu

Co-Investigator
Jed Rose, Ph.D.
jed.rose@duke.edu

Statistician
Frances Wang
frances.wang@duke.edu

Study Coordinator
Leah Thomas, MS, CRC, LRT/CTRS
leah.thomas@duke.edu

Version Date: 02/26/2020

1 Table of Contents

2 LIST OF ABBREVIATIONS 5

3 PROTOCOL SYNOPSIS AND RESEARCH SUMMARY 6

3.1 Purpose 6

3.2 Background and Significance 7

3.3 Design and Procedure 10

3.4 Selection of Subjects 10

3.5 Duration of study 11

3.6 Data Analysis and Statistical Considerations 11

4 Study Schema 12

5 BACKGROUND AND SIGNIFICANCE 13

5.1 Study Condition 13

5.2 Study Drug 13

5.2.1. Pre-clinical experience of study drug 15

5.2.2. Clinical experience 16

5.3 Study Purpose/Rationale 16

6 OBJECTIVES AND ENDPOINTS 17

7 INVESTIGATIONAL PLAN 19

7.1 Study Design 19

7.1.1. Dose Modification 19

7.1.2. Safety Considerations 19

7.1.3. Missed Doses 19

7.1.4. Concomitant Medications/Therapies 19

7.1.5. Study Drug Blinding 19

7.1.6. Randomization 19

7.2 Rationale for Selection of Dose, Regimen, and Treatment Duration 19

7.3 Rationale for Correlative Studies 20

7.4 Definition of Evaluable Subjects, On Study, and End of Study 20

7.5 Early Study Termination 20

8 STUDY DRUG 20

8.1 Names, Classification, and Mechanism of Action 20

8.2 Packaging and Labeling 20

8.3 Supply, Receipt, and Storage 21
8.4 Dispensing and Preparation 21
8.5 Compliance and Accountability 21
8.6 Disposal and Destruction 21
9 SUBJECT ELIGIBILITY 21
9.1 Inclusion Criteria 21
9.2 Exclusion Criteria...21
10 TRIAL PROCEDURES AND ASSESSMENTS 23
10.1 Screening Examination...24
10.2 Treatment Period 25
10.3 End of Treatment 27
10.4 Follow-up Period 27
10.5 End of Study 27
10.6 Early Withdrawal of Subject(s)..28
10.6.1. Criteria for Early Withdrawal 28
10.6.2. Follow-up Requirements for Early Withdrawal 28
10.6.3. Replacement of Early Withdrawal(s) 28
10.7 Study Assessments..28
10.7.1. Medical History 28
10.7.2. Physical Exam...28
Subject-Rated Non-Standardized Measures 28
10.7.3. Subject-Rated Standardized Measures 29
10.7.4. Subject-Rated Non-Standardized Measures to Be Collected at All Clinic Visits 29
10.7.5. Subject-Rated Standardized Measures to Be Collected at All Clinic Visits 29
10.7.6. Subject-Rated Measures to Be Collected through Phone Assessment 29
11 SAFETY MONITORING AND REPORTING 29
11.1 Adverse Events..29
11.1.1. AEs of Special Interest...30
11.1.2. Reporting of AEs...30
11.2 Serious Adverse Events 30
11.2.1. Reporting of SAEs..30
11.3 Emergency Unblinding of Investigational Treatment 31
11.4 Other Reportable Information 31
11.5 Special Warnings and Precautions...31

11.6 Stopping Rules 31
11.7 Safety Oversight Committee (SOC) 32
11.8 External Data and Safety Monitoring Board (DSMB) 32
12 QUALITY CONTROL AND QUALITY ASSURANCE 32
12.1 Monitoring 32
12.2 Audits 32
12.3 Data Management and Processing 33
12.3.1. Case Report Forms (CRFs) 33
12.3.2. Data Management Procedures and Data Verification 33
12.3.3. Study Closure 33
13 STATISTICAL METHODS AND DATA ANALYSIS 33
13.1 Analysis Sets 32
13.2 Patient Demographics and Other Baseline Characteristics 33
13.3 Treatments 33
13.4 Primary Objective 33
13.4.1. Variable 33
13.4.2. Statistical Hypothesis, Model, and Method of Analysis 33
13.4.3. Handling of missing values, censoring, and discontinuations 33
13.5 Secondary Objectives 33
13.5.1. Key Secondary Objective 33
13.5.2. Other Secondary Objectives 33
13.6 Exploratory Objectives 35
13.6.1. Key Exploratory Objective 35
13.6.2. Other Exploratory Objectives 35
13.7 Interim Analyses 36
13.8 Sample Size Calculation 36
14 ADMINISTRATIVE AND ETHICAL CONSIDERATIONS 37
14.1 Regulatory and Ethical Compliance 37
14.2 DUHS Institutional Review Board and DCI Cancer Protocol Committee 37
14.3 Informed Consent 37
14.4 Study Documentation 37
14.5 Privacy, Confidentiality, and Data Storage 38
14.6 Data and Safety Monitoring 38
14.7 Protocol Amendments 38

14.8 Records Retention....38
15 REFERENCES....39
16 APPENDICES....43
16....57

2 LIST OF ABBREVIATIONS

AE	Adverse event
AUDIT-C	Alcohol Use Disorders Identification Test
BID	Twice a day
CO	Carbon monoxide
CPD	Cigarettes per day
CRC	Clinical Research Coordinator
CSC	Center for Smoking Cessation
CTA	Clinical Trials Assistant
DSCP	Duke Smoking Cessation Program
DSMB	Data and Safety Monitoring Board
DUHS	Duke University Health System
EMR	Electronic medical record
FDA	Food and Drug Administration
FTND	Fagerström Test of Nicotine Dependence
GAD-2	Generalized Anxiety Disorder 2-item Scale
GCP	Good Clinical Practice
ICS	Investigational Chemotherapy Service
IRB	Institutional Review Board
NIDA	National Institute on Drug Abuse
NIH	National Institutes of Health
MPSS	Mood and Physical Symptoms Scale
P	Placebo (e.g. P-Varenicline is Placebo Varenicline)
PHI	Protected Health Information
PHQ-9	Patient Health Questionnaire-9
PSS-4	Perceived Stress Scale
SAE	Serious adverse event
TQD	Target Quit Day

3 PROTOCOL SYNOPSIS AND RESEARCH SUMMARY

3.1 Purpose

The purpose of this study is to assess an “adaptive” approach to smoking cessation pharmacotherapy. The protocol is designed to compare adaptive vs. standard approaches to two common smoking cessation pharmacotherapies (Varenicline and Nicotine Patch). The adaptive treatment approach provides the addition of Bupropion in the pre-quit period for participants who are not “responding” to initial treatment. Little is known about the adaptive use of Varenicline or Nicotine Patch, in which Bupropion is added to Varenicline or Patch for those who do not respond to one of these medications in a pre-quit treatment period. This study attempts to address these knowledge deficits. The study (N=300) is a double-blinded randomized placebo-controlled trial designed to compare biochemically-confirmed abstinence rates in smokers randomized to Varenicline Adaptive Protocol vs. Varenicline (N=150) and for comparison, Nicotine Patch Adaptive Protocol vs. Nicotine Patch (N=150). The “Varenicline Adaptive Protocol” is conducted by starting treatment with Varenicline 4 weeks prior to the quit day, then following each participant’s response to this pre-treatment medication. After 2 weeks, if the patient shows a reduction greater than 50% in cigarettes smoked per day then the patient is considered to be a “Varenicline responder” and is continued on Varenicline alone out to 12-weeks post quit day. If the patient does not spontaneously decrease smoking in the pre-quit period by more than 50% cigarettes per day, the patient is considered to be a “Varenicline non-responder” and Bupropion is added to the Varenicline. For comparison, an identical protocol is used with nicotine patch vs. nicotine patch adaptive treatment. The study uses only FDA-approved medications: Varenicline, Nicotine Patch, Bupropion, and placebo controls. To pattern clinical practice, participants will be able to choose whether they would like to use a patch or Varenicline-based treatment. After choosing, however, they will be randomized to adaptive vs. non-adaptive version of that treatment. Placebo medications are matched throughout the study. Participants will be blinded to all medications. All participants will receive behavioral treatment including a single 40-minute visit with a medical provider. The study is designed to provide researchers and clinicians with a better understanding of how to use adaptive pharmacotherapy protocols to improve smoking cessation rates.

Primary Objective

1. Biochemically-confirmed 30-day continuous smoking abstinence at 12-weeks post quit attempt measured by self-report and confirmed by carbon monoxide (CO) expired breath testing with CO < 7ppm required to confirm abstinence. Subject will be coded as abstinent only if he or she meets criteria for both self-reported abstinence and biochemical abstinence.

Secondary Objectives

2. To compare 7-day point prevalence biochemically confirmed abstinence rates at 2- and 12-weeks post-TQD in participants randomized to adaptive vs standard treatment.
3. To compare phone-assessed self-reported abstinence at 1, 2, 6, 12, 26 and 52 weeks post-TQD in participants randomized to adaptive vs standard treatment. Phone assessments will be compared at specific time points and also across multiple time points using multi-level modeling.
4. To compare smoking reduction (number of cigarettes per day) at 2 and 12 weeks post TQD in participants randomized to adaptive vs standard treatment.
5. To compare smoking reduction (number of cigarettes per day) on phone-based self-report at 1, 2, 6, 12, 26, and 52 weeks post-TQD in participants randomized to adaptive vs. standard treatment. Phone assessments will be compared at specific time points and also across multiple time points using multi-level modeling.

Exploratory Objectives

6. To assess potential baseline (pre-intervention) predictors of abstinence including self-reported dependence, stress, anxiety, depression, alcohol and drug use, and self-efficacy. If a baseline variable is found to be associated with abstinence, it will be further assessed as a moderator of abstinence.
7. To assess change in self-reported measures at baseline (pre-quit) to 2- and 12-weeks post TQD on repeated standardized measures of nicotine dependence, stress, anxiety, depression, alcohol and drug use, and self-efficacy. If a measure is found to change significantly from baseline to a post quit time point, it will be assessed as possible mediator of abstinence outcomes.
8. To compare phone-assessed non-standardized single item questions on urges, withdrawal, motivation to quit smoking or remain abstinent, and confidence in ability to quit or remain abstinent at 1, 2, 6, 12, 26 and 52 weeks post-TQD. If a measure is found to change significantly from baseline to a post quit time point, it will be assessed as a possible mediator of abstinence outcomes. Phone assessments will be compared at specific time points and also across multiple time points using multi-level modeling.

9. To compare baseline scores on anxiety, depression, and stress for their effect on allocation to Responder or Non-Responder status in the adaptive and standard treatment arms.

10. To conduct cost-benefit analyses on allocation to adaptive vs standard treatment from the perspective of multiple stake holders: smokers, healthcare systems (clinics/hospitals), insurance companies, and employers.

Hypotheses

This protocol is hypothesis-supporting and hypothesis-testing.

Primary Hypothesis:

1. Participants allocated to adaptive therapy will show significantly higher biochemically confirmed 30-day continuous abstinence at 12 weeks post TQD.

Secondary Hypotheses:

2. Participants allocated to adaptive vs standard treatment will show significantly higher biochemically confirmed 7-day point prevalence abstinence at 2 and 12 post TQD assessment visits.
3. Participants allocated to adaptive vs standard treatment will show significantly higher phone-assessed self-reported abstinence at 1, 2, 6, 12,26, and 52 weeks post-TQD.
4. Participants allocated to adaptive vs standard treatment will show lower number of cigarettes per day (cpd) at 2- and 12-weeks post TQD.
5. Participants allocated to adaptive vs standard treatment will show lower number of cigarettes per day (cpd) on phone-based self-report at 1, 2, 6, 12, 26, and 52 weeks post-TQD.

Exploratory Objectives

6. The following baseline self-report assessment outcomes will predict biochemically confirmed 30-day continuous abstinence at 12 weeks post TQD and 7-day point prevalence abstinence at 2- and 12-weeks post-quit: nicotine dependence, depression, anxiety, stress, alcohol use, drug use, motivation, and self-efficacy.
7. The following baseline self-report assessment outcomes will predict lower number of cpd and higher number of days abstinent at 2- and 12- week visits: urge, withdrawal symptoms, depression, anxiety, stress, alcohol use, drug use, motivation, and self-efficacy.
8. The following baseline self-report assessment outcomes will predict abstinence on phone-based self-report at 1, 2, 6, 12, 26, and 52 weeks post-TQD urges, withdrawal, motivation to quit smoking or remain abstinent, and confidence in ability to quit or remain abstinent
9. Baseline scores on anxiety, depression, and stress will moderate the effect of allocation to Non-Responder status in the adaptive and standard treatment arms. This finding will show that poor response to pharmacotherapy may be related to high levels of anxiety, depression, or stress.
10. A prospective economic analysis of adaptive vs standard treatment will show cost-benefit outcomes from the perspective of people that smoke cigarettes, healthcare systems, insurance companies, and employers.
11. **Feasibility:** Demographics of patients at DSCP and Duke CSC will reflect the recruitment area
12. **Feasibility:** Medications used will show wide variation of use.
13. **Feasibility:** Attendance at DSCP Return Appointments will be favorable.
14. **Feasibility:** Referral Routes to DSCP will reflect recruitment area.
15. **Feasibility:** Medication side effects will be favorable.
16. **Feasibility:** Medication adherence will be favorable.

3.2 Background and Significance

Adaptive Treatment Regimens: The primary study treatment is a medication strategy referred to here as the adaptive pharmacotherapy algorithm. This strategy uses FDA-approved medications for smoking cessation (Varenicline, Nicotine Patch and Bupropion). Two weeks after starting these treatments, participants are assessed to see how much they have reduced their smoking on these medications. If, after 2 weeks of treatment, participants have reduced their cpd to 50% or less of baseline, they are considered to be "responsive" to treatment and are continued on this treatment alone until 12 weeks post-TQD. After 2 weeks of treatment, participants who have not decreased their cigarette use to 50% or more of their baseline cpd are considered "non-responders", and Bupropion is added to the treatment regimen 2 weeks prior to the quit attempt and continued until 12 weeks post TQD. Estimates based on prior studies within our lab (see preliminary studies) suggest 50% will be non-responders.

Pharmacotherapy Control: The control group used for this algorithm is Varenicline started 1 week prior to TQD (1 week escalating dose) or Nicotine Patch started on the TQD and continuing to 12 weeks post-TQD. This is considered standard of care treatment. P-Varenicline and P-Patch for control participants only will be started 4 weeks prior to TQD, and P-Bupropion will be started 1 week prior to TQD to match study groups

Behavioral Treatments: All participants will receive one 40-minute visit with a medical provider who specializes in tobacco dependence treatment.

Rationale for Research: The primary disease population is people that smoke cigarettes, with the goal of decreasing the incidence of cancer and other smoking-related illnesses. Smokers are at significantly increased risk of multiple forms of cancer (USDHHS, 2014). The unmet need of this treatment is that current methods used to treat smokers yield low abstinence rates. Smoking is the number one cause of preventable morbidity and mortality in the US, claiming over 500,000 lives per year (USDHHS, 2014). There is currently compelling evidence from our research center (Duke Center for Smoking Cessation) and from other investigators that adaptive treatment of smokers who fail to respond to Varenicline or Nicotine patch alone will lead to higher abstinence rates than Varenicline used in usual dosing protocols (see below). If the adaptive approach is found to be effective, it might be implemented as a treatment strategy by clinicians.

Existing Evidence for Intervention

A. Use of Adaptive Pharmacotherapy Algorithm: Trials have shown that a number of pharmacotherapies are effective for smoking cessation including Bupropion, Nicotine Lozenge, and Nicotine Patch and have found even greater effectiveness for combination nicotine therapy and Varenicline (Mills et al., 2012; Piper et al., 2009). Today, however, there is growing evidence that pharmacotherapies might be more effective if they are tailored to specific patients based on their early response to a treatment regimen (Pancani et al., 2015). Preliminary studies at Duke University have demonstrated a simple method to provide "targeted therapy" to smokers through an adaptive pharmacotherapy algorithm, referred to throughout this protocol as the "Algorithm"(Rose, 2011; Rose & Behm, 2013, 2014; Rose, Herskovic, Behm, & Westman, 2009). Algorithm-based treatment results show two patterns of response. Responders (smokers who decrease their cigarette use by 50% or more during the pre-quit treatment) show high-level abstinence rates using only the medication on which they were started (see preliminary studies below). Non-Responders (smokers who do not show a 50% decrease in cigarette use during the pre-quit treatment) show much lower abstinence rates overall (see below) with Patch or Varenicline but show significant increases in abstinence rates with the addition of Bupropion. In this study, we will provide non-responders with Bupropion as an "adaptive treatment" with the goal of improving abstinence rates in these individuals.

B. Use of Behavioral Therapies: It is now well established that the use of pharmacotherapies and behavioral therapies together result in higher abstinence rates than either approach alone (Alterman, Gariti, & Mulvaney, 2001; Evins et al., 2001; Fiore, 2008; Whitlock, Orleans, Pender, & Allan, 2002). Meta-analyses across multiple interventions show that the effectiveness of behavioral therapy is robustly related to treatment intensity and overall time commitment (Fiore, 2008; Stead & Lancaster, 2012). In accordance with these findings, the study will utilize a single 40-minute treatment with encouragement, quit day planning, discussion of prior quit attempts, and smoking triggers.

C. Economic Assessment: Economic analyses have found smoking cessation interventions to be highly cost-effective when compared to conventional standards of quality-adjusted life year saved (QALYs), including sub-populations of older smokers, pregnant women, and racial/ethnic minorities (Barnett et al., 2014; Cooper et al., 2014; Dornelas et al., 2006; Gordon, Graves, Hawkes, & Eakin, 2007; Ronckers, Groot, & Ament, 2005; Ruger & Lazar, 2012; Ruger, Weinstein, Hammond, Kearney, & Emmons, 2008). There is growing evidence that the financial gains to employers from employee smoking cessation may make employer-based cost incentives cost-effective to both employers and employees (Berman, Crane, Seiber, & Munur, 2014; Bunn, Stave, Downs, Alvir, & Dirani, 2006; Burton et al., 2005; Giles, Robalino, McColl, Sniehotta, & Adams, 2014; Halpern, Shikiar, Rentz, & Khan, 2001). With development of new treatment strategies, there is a growing need for studies testing the economic impact of outcomes in healthcare systems (Papadakis et al., 2014). The proposed study includes an economic analysis of adaptive treatment vs. standard pharmacotherapy.

Preliminary Studies

A. Pre-Treatment Response Delineates Smokers into Groups with Differing Treatment Outcomes: Several studies have found an association between pre-quit smoking reduction and subsequent smoking abstinence (Papadakis et al., 2014; Rose, 2011; Rose & Behm, 2013, 2014; Rose et al., 2009). In one study, providing 13 weeks of nicotine patch therapy with quit day at week 2, 53.8% of the smokers placed on a nicotine patch decreased their cigarette use by 50% within 1 week (Rose & Behm, 2013). These "responders" showed significantly higher biochemically confirmed 4-week continuous abstinence at 11 weeks post-quit (p = 0.0006, *OR* = 2.3, 95% CI = 1.43-3.72) compared to "Non-Responders," who did not decrease their cigarette use by 50% after 1 week of pre-treatment. Nicotine Patch response was assessed via cigarette use at week-1 and used to determine which testing method allocated subjects in such a way as to yield the highest abstinence. Other studies (Ebbert, 2013) have shown similar responses to pre-quit Varenicline, in which rates of smoking during the pre-quit period are associated with long-term abstinence. The current study design is drawn from several studies conducted at the Duke Center for Smoking Cessation as well as studies by others. Conclusions drawn from prior NIDA P50-funded studies initially demonstrated the usefulness of "adaptive treatment" (adding bupropion for non-responders to pre-quit treatment).

B. Efficacy of Adaptive Treatment:

In this study, 50% of smokers will likely be classified as treatment responders. In a comparison across similar studies, treatment responders demonstrated 11-week post-quit 4-week continuous abstinence on nicotine patch at 39.0% and 36.9%, and 63.9% (p = 0.018) on Varenicline (Rose & Behm, 2013, 2014; Rose et al., 2009). Varenicline alone was not significantly different from Varenicline + Bupropion at 70.0% (p = 0.99) (Rose & Behm, 2014). In another P50 Study-4, pre-treatment Non-Responders demonstrated biochemically confirmed 11-week post-quit, 4-week continuous abstinence rate for: Varenicline + Bupropion = 44.4% vs. Varenicline alone = 18.6% (p = 0.001, *OR* = 3.51, 95% CI = 1.64-7.51).This finding is supported by a recent Mayo Clinic study in which smokers achieved prolonged 12-week post-quit abstinence with Varenicline alone at 34.2% vs. Varenicline + Bupropion at 47.5%, (p = 0.04, *OR* = 1.74, 95% CI = 1.04-2.93). If they are "treatment responders" they do not benefit significantly from the addition of Bupropion to Varenicline, but if they are non-responders, the addition of Bupropion appears to significantly improve abstinence rates, supporting the use of adaptive pharmacotherapy for Varenicline. Regarding use of the nicotine patch, we found that smokers did well using nicotine patch *if* they reduced their cpd by 50% during pre-quit treatment. Analysis of these "treatment responders" demonstrated biochemically-confirmed 4-week continuous abstinence at 11 weeks post-quit attempt of 54.2% in one study (n = 71) and 59.0% in another study (n = 39) (Rose & Behm, 2013, 2014). On the other hand, treatment non-responders showed 11-week post-quit attempt biochemically confirmed 4-week continuous abstinence at the following rates: Patch = 16.8% (n = 45), vs. Patch + Bupropion = 39.5% (n = 38) (p = 0.03, *OR* = 3.0, 95% CI = 1.1-8.2), vs. Varenicline = 19.0% (n = 42) (p = 0.88, *OR* = 1.09, 95% CI = 0.37-3.2). This data suggests that it is reasonable to start smokers who choose the nicotine patch with just the patch, and then add Bupropion if they do not reduce cpd by 50% (Rose & Behm, 2014).

Application of Results

1. Algorithm Testing: The Algorithm developed through the P50 has not been tested as a whole. Prior P50 funded studies have assessed the general principle of adaptive treatment (as above) and tested individual quadrants of the Algorithm. The proposed study is designed to test the Algorithm as a whole within a double-blinded randomized placebo-controlled trial. If findings are positive, it will influence the use of algorithm-based treatment and longer treatment periods: 4-week pre-treatment period with adaptive assessment, followed by 12 weeks of post-TQD treatment.

2. Assessment of Sub-Populations: There is a growing body of evidence that smokers are a heterogeneous population, with variation in treatment response dependent on individual characteristics (Cahill & Perera, 2008; Fiore, 2008; Shiffman, Sweeney, & Dresler, 2005; Sussman, Valente, Rohrbach, Skara, & Pentz, 2006; Wagena, Knipschild, Huibers, Wouters, & van Schayck, 2005; Wagena, van der Meer, Ostelo, Jacobs, & van Schayck, 2004). The study design includes an analysis of baseline characteristics – dependence, depression, anxiety, and stress that show evidence as predictors of relapse.

3. Economic Assessment: Economic barriers must always be considered in the successful "real-world" implementation of smoking cessation therapies (Eddy, 2009; Fiore, 2008; Nides, Leischow, Sarna, & Evans, 2007; Warner, Mendez, &

Smith, 2004). An economic analysis will be conducted to generate a prospective economic analysis of adaptive vs. standard pharmacotherapy treatment to identify cost-benefit outcomes from the perspective of smokers, healthcare systems, insurance companies, and employers.

3.3 Design and Procedure

This is an investigational study (n=300) designed to test a Varenicline and Nicotine Patch-based adaptive pharmacotherapy algorithm. The study is designed to compare two smoking cessation pharmacotherapy strategies: Adaptive vs. standard treatment. Individual arms of this adaptive algorithm have demonstrated compelling results, but the algorithm itself has not been studied as an integrated treatment tool.

CHOICE OF VARENICLINE VS. PATCH THEN RANDOMIZATION TO ADAPTIVE VS. STANDARDTREATMENT

N=300	RANDOMIZATION WITHIN PARTICIPANTS TO ADAPTIVE VS. STANDARD PROTOCOLS	
CHOICE:<br>VARENICLINE-BASED RX<br>(n=150)	VARENICLINE ADAPTIVE PROTCOL<br>(N=75)	VARENICLINE STANDARD PR0TOCOL<br>(N=75)
CHOICE:<br>PATCH-BASED RX<br>(n=150)	NICOTINE PATCH ADAPTIVE PROTOCOL<br>(N=75)	NICOTINE PATCH STANDARD PROTOCOL<br>(N=75)

ADAPTIVE PHARMACOTHERAPY ALGORITHM (n=300)

**VARENICLINE-BASED TREATMENT**<br>Participant choses Varenicline-based treatment and is then randomized to Adaptive Treatment arm (N=75)	**NICOTINE PATCH-BASED TREATMENT**<br>Participant choses Nicotine patch-based treatment and is then randomized to Adaptive Treatment arm (N=75)
**VARENICLINE NON-RESPONDER**<br>4 weeks prior to TQD: Start Varenicline<br>2 weeks prior to TQD, DOES NOT reduce cigs/day by > 50%<br>1 week prior to TQD: Start Bupropion<br>Use Varenicline + Bupropion to 12 weeks post TQD	**NICOTINE PATCH NON-RESPONDER**<br>4 weeks prior to TQD: Start Nicotine Patch<br>2 weeks prior to TQD, DOES NOT reduce cigs/day by > 50%<br>1 week prior to TQD: Start Bupropion<br>Use Nicotine Patch + Bupropion to 12 weeks post TQD
**VARENICLINE RESPONDER**<br>4 weeks pre-TQD: Start Varenicline<br>2 weeks prior to TQD - DOES reduce cigs/day by > 50%<br>1 week prior to TQD: Start Placebo Bupropion<br>Use Varenicline + Placebo Bupropion to 12 weeks post TQD	**NICOTINE PATCH RESPONDER**<br>4 weeks pre-TQD: Start Nicotine Patch<br>2 weeks prior to TQD - DOES reduce cigs/day by > 50%<br>1 week prior to TQD: Start Placebo Bupropion<br>Use Nicotine Patch + Placebo Bupropion to 12 weeks post TQD

STANDARD PHARMACOTHERAPY PROTOCOL:

**VARENICLINE-BASED TREATMENT**<br>Participant choses Varenicline-based treatment and is then randomized to Standard Treatment arm (N=75)	**NICOTINE PATCH-BASED TREATMENT**<br>Participant choses Nicotine patch-based treatment and is then randomized to Standard Treatment arm (N=75)
**VARENICLINE STANDARD TREATMENT**<br>4 weeks pre-TQD: Start Placebo Varenicline<br>1 week prior to TQD – Switch to Active Varenicline<br>1 week prior to TQD: Start Placebo Bupropion<br>Use Varenicline + Placebo Bupropion to 12 weeks post TQD	**NICOTINE PATCH STANDARD TREATMENT**<br>4 weeks pre-TQD: Start Placebo Nicotine Patch<br>TQD - start Nicotine Patch<br>1 week prior to TQD: Start Placebo Bupropion<br>Use Nicotine Patch + Placebo Bupropion to 12 weeks post TQD

3.4 Selection of Subjects

This study will recruit participants through Quit at Duke, also called the Duke Smoking Cessation Program (DSCP), the Duke Primary Care Research Consortium, and the Duke Center for Smoking Cessation (CSC). The DSCP is a growing multi-clinic tobacco use treatment program. The Duke CSC is a research center dedicated to researching novel

treatments to help smokers break their addiction to nicotine. Subjects for this study will be required to be current smokers, confirmed by self-report and carbon monoxide testing (CO over 7) who are at least 18 years of age. Subjects additionally must also be willing to set a Quit Day (day they intend to stop smoking) within the next six weeks and be willing to use two medications together – either Varenicline and Bupropion or Nicotine Patch and Bupropion depending on their choice of regimen. Subjects will be excluded if they are currently pregnant or breast feeding, test positive for drug or alcohol abuse psychometrically, use a secondary form of tobacco or nicotine, are already taking a tobacco use treatment medication, have a symptomatic mental or physical illness, or are unwilling to engage in pharmaceutical and behavioral treatments for tobacco use.

3.5 Duration of study

All subjects will receive pharmacotherapy +/- placebo treatment for 16 weeks (4 weeks pre-TQD, then 12 weeks post-TQD). Subjects will be called for brief phone assessments up to 12-months post TQD. Total study duration will be approximately 13 months.

3.6 Data Analysis and Statistical Considerations

Detailed Data Analysis is provided in Section 13. A summary of data analysis procedures includes the following: **Multilevel Modeling:** Given repeated observations across multiple individuals, many of the analyses in this study will be derived from multilevel modeling (MLM). **Missing Data:** Missing data will be examined to determine whether missingness is systematic (i.e., associated with individual baseline differences or time-related changes in observed variables). An intent-to-treat approach in which subjects lost to follow-up will be counted as non-abstinent will also be used to analyze univariate outcomes. An alpha criterion of 0.05 (two-tailed) will be used in all comparisons. **Analysis of Primary Outcome:** The primary study outcome is comparison of abstinence rates in adaptive vs standard treatment groups. The primary outcome measure is biochemically confirmed 30-day continuous abstinence at the 12-week post-quit attempt (carbon monoxide breath testing with CO < 7ppm). **Secondary abstinence outcomes**: Includes comparison of abstinence rates in adaptive vs standard treatment groups via self-reported 7 and 30-day continuous abstinence at 2- and 12-weeks post-TQD as well as phone-based self-reported abstinence at 1, 2, 6, 12, 26, and 52 weeks post-TQD. **Continuous Smoking Outcomes:** In addition to dichotomous outcomes described above, there will be an assessment of number of cpd and number of days abstinent at 2- and 12-week post TQD assessment visit (as well as phone assessment time points). **Assessment of potential moderators of primary effect:** The following constructs will be assessed as potential predictors of abstinence: demographics (age, gender, race, education), smoking history and heaviness, prior quit attempts, financial status, dependence (FTND), urge rating (MPSS), depression (PHQ-2), anxiety (GAD-2), alcohol use (AUDIT-C), stress (PSS-4), self-efficacy questions. **Assessment of potential mediators of primary effect:** The following constructs will be assessed to determine whether assignment to pharmacotherapy group is associated with change in these constructs pre- and post-treatment: urge rating (MPSS), depression (PHQ-2), anxiety (GAD-2), stress (PSS-4), self-efficacy. **Economic Analysis:** An economic analysis of the Algorithm vs. Standard treatment from multiple perspectives will be provided. **Sample Size Calculation:** Total sample size required to show differences on primary outcomes between pharmacotherapies and behavioral treatments with 10% downward adjustment in study group outcomes for possibility of unexpectedly poor outcomes is **N = 300.**

4 Study Schema

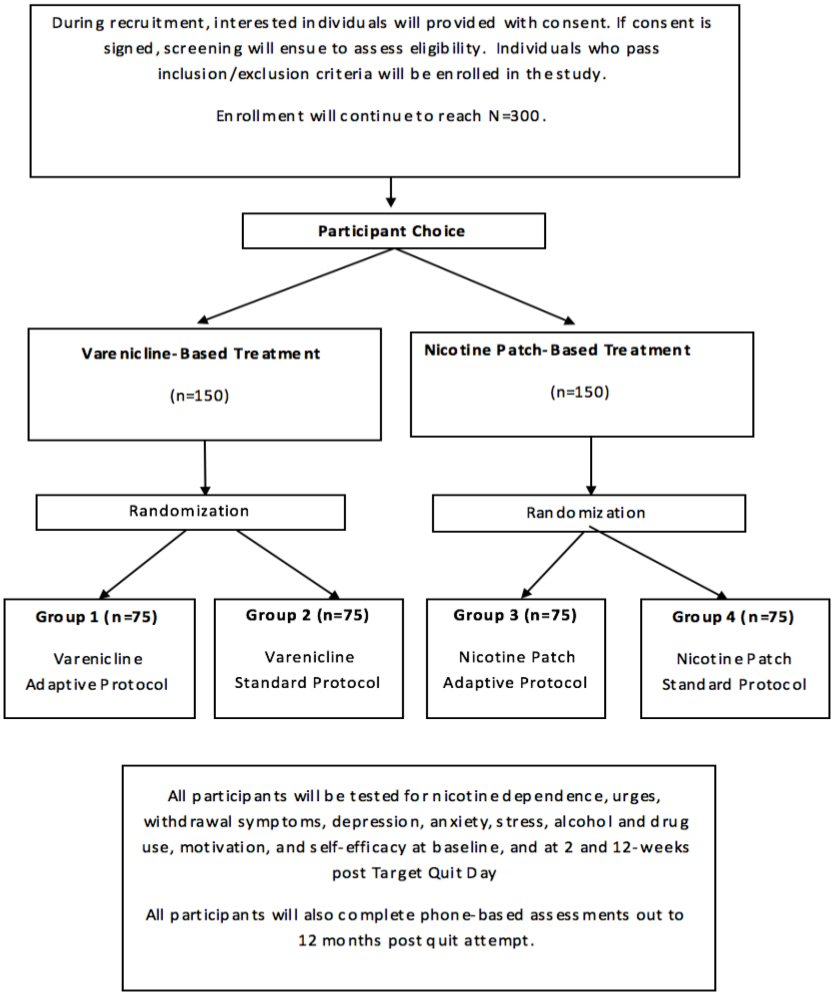

5 BACKGROUND AND SIGNIFICANCE

5.1 Study Condition

Tobacco use is the number one cause of preventable morbidity and mortality in the US, has a profound economic impact nationally, and devastating health effects globally (CDC, 2008; USDHHS, 2014; USSGsACo, 1964; WHO, 2002). The contemporary person that smokes cigarettes has greater nicotine dependence, resistance to available therapies, and increased need for new, more effective tobacco use treatment therapies (Brandon, 2001; Cokkinides et al., 2009; Fiore, 2008; J. Hughes, Lindgren, Connett, Nides, & Lung Health, 2004; Irvin, Hendricks, & Brandon, 2003; Orleans, 2007; Warner & Burns, 2003) Trials have shown that a number of pharmacotherapies are effective for smoking cessation including bupropion, nicotine lozenge, and nicotine patch and have found even greater effectiveness for Varenicline (Mills et al., 2012; Piper et al., 2009). There is, however, limited evidence on the use of Varenicline or Nicotine patch within adaptive pharmacotherapy algorithms.

5.2 Study Drug

There are three smoking cessation medications used in this study: Nicotine Patch, Varenicline and Bupropion. All are FDA-approved for use as pharmacotherapy for tobacco use treatment. Below is information on each including mechanism of action, rational for use and potential side effects.

Varenicline (Chantix)

Mechanism of Action: Varenicline stimulates nicotinic receptors (partial agonist at α4β2 Nicotinic receptor and full agonist at α7) and provides some relief from withdrawal symptoms related to empty receptors with half-life of over 12 hours (Taly, Corringer, Guedin, Lestage, & Changeux, 2009). The sustained stimulation of nicotinic receptors has a similar effect to that of the nicotine patch. Varenicline does a bit more however: When it binds to a nicotinic receptor, it also blocks that receptor from nicotine so that when a person lapses and smokes a cigarette after their quit day, the nicotine does not bind to blocked nicotinic receptors. Because some receptors remain open for nicotine, smoking does have an effect, but a diminished one. While using Varenicline, people often find that it is not satisfying to smoke. This helps a person return to abstinence after a lapse. Additionally, Varenicline binds nicotinic receptors differently than nicotine. There are a variety of nicotinic acetylcholine receptors; α4β2 - 4 alpha subunits, and 2 beta subunits are most common. However, α7, α3β4, α3β2, α6 and others play a role in nicotine dependence (Sharples, 2001). Varenicline is a partial agonist at the α4β2 receptor, but is a strong and full agonist at α7, now thought to be very important to dependence (Mihalak, Carroll, & Luetje, 2006).
Rationale for Use: Abstinence rates for Varenicline compared to placebo are OR = 2.88. Because the abstinence rate is so high for Varenicline compared to other treatments, Varenicline is considered standard-of-care treatment. The main problem with Varenicline is a relatively large number of patients have intolerable side effects; however, most tolerate it well (Cahill, Stevens, Perera, & Lancaster, 2013).

Dosing (Pfizer, 2014)
Begin Chantix 1 week before "Quit Day". Total duration of treatment is 12 weeks (Pfizer, 2014)(Pfizer, 2014)(Pfizer, 2014)
Days 1-3: 0.5mg once daily (total dose 0.5mg/day)
Days 4-7: 0.5mg twice daily (total dose 1mg/day)
Day 8 till end of treatment: 1mg twice daily (total dose 2mg/day)
Further instructions for use: Take with a full glass of water in order to minimize risk of nausea

Contraindications: In patients with severe renal impairment, dosing of Varenicline needs to be altered. Varenicline is not recommended for use in pregnant or breastfeeding women and in patients <18 years (Fagerstrom & Hughes, 2008).

Side effects (Fagerstrom & Hughes, 2008):
Nausea: 17 - 44% (attributable discontinuation rates between 0.6 – 0.7%)
Insomnia: 5.1 – 42.9%
Abnormal dreams: 5.6 – 37.2%
Headache: 5.5 – 24.2%
Other side effects include gastrointestinal disturbances such as constipation and flatulence.
In individuals with pre-existing psychiatric conditions there is a slightly increased incidence of anxiety and mood related disorders. However, important new data shows that there is no increased risk of suicidal behavior in individuals taking

Varenicline (Molero, Lichtenstein, Zetterqvist, Gumpert, & Fazel, 2015). Studies have shown that individuals who are treated with Varenicline have a 1.5 greater chance of discontinuing Varenicline compared to those on placebo (Drovandi, Chen, & Glass, 2016).

<u>Nicotine Patch</u>

Mechanism of Action: Nicotine from cigarette smoke causes dependence by working as an agonist at nicotinic acetylcholine receptors (nAChRs). When nAChRs are activated they release different types of neurotransmitters depending on the receptor that was activated. When nicotine binds to α4β2 nAChRs it increases the release of dopamine which has effects within the reward and addiction centers in the brain. Nicotine patch work as agonists of nAChR type α4β2 therefore stimulating the release of dopamine to reduce nicotine withdrawal symptoms.

Rationale for Use: Nicotine patch decreases symptoms of physical withdrawal and eliminates acute reinforcing effects of nicotine so that patients are able to concentrate their efforts on behavioral and psychological components of becoming tobacco free. Nicotine patch is now the cornerstone of tobacco use treatment pharmacotherapy. Patients generally tend to be more compliant with nicotine patches than other FDA-approved nicotine replacement treatment medications as the patch requires dosing only once a day and because they are the least conspicuous to administer. Added benefits are that nicotine patches deliver consistent levels of nicotine during a 24-hour period and can be used with other smoking cessation pharmacotherapies.

Dosing: Dosing is variable depending on cpd:
EXAMPLE Dosing for 12 weeks of treatment: Take one 21 mg Patch per day for 8 weeks, then one 14 mg patch per day for 2 weeks, then one 7 mg patch for 2 weeks. Tapering however is optional and is not evidence-based. Further instructions for use: Patch can be worn 24 hours per day and then move the site of the patch each morning. If the patient experiences insomnia or disturbing dreams from wearing a patch at night, the patient can remove the patch before sleep.

Common Side effects: Only 4% of smokers stop using the transdermal nicotine patch due to adverse effects, a lower rate than is found in other smoking cessation medications. The most commonly reported adverse effects for those who quit using patch due to a side effect are provided in the table below by Mills, Wu, Lockhart, Wilson, and Ebbert (2010).

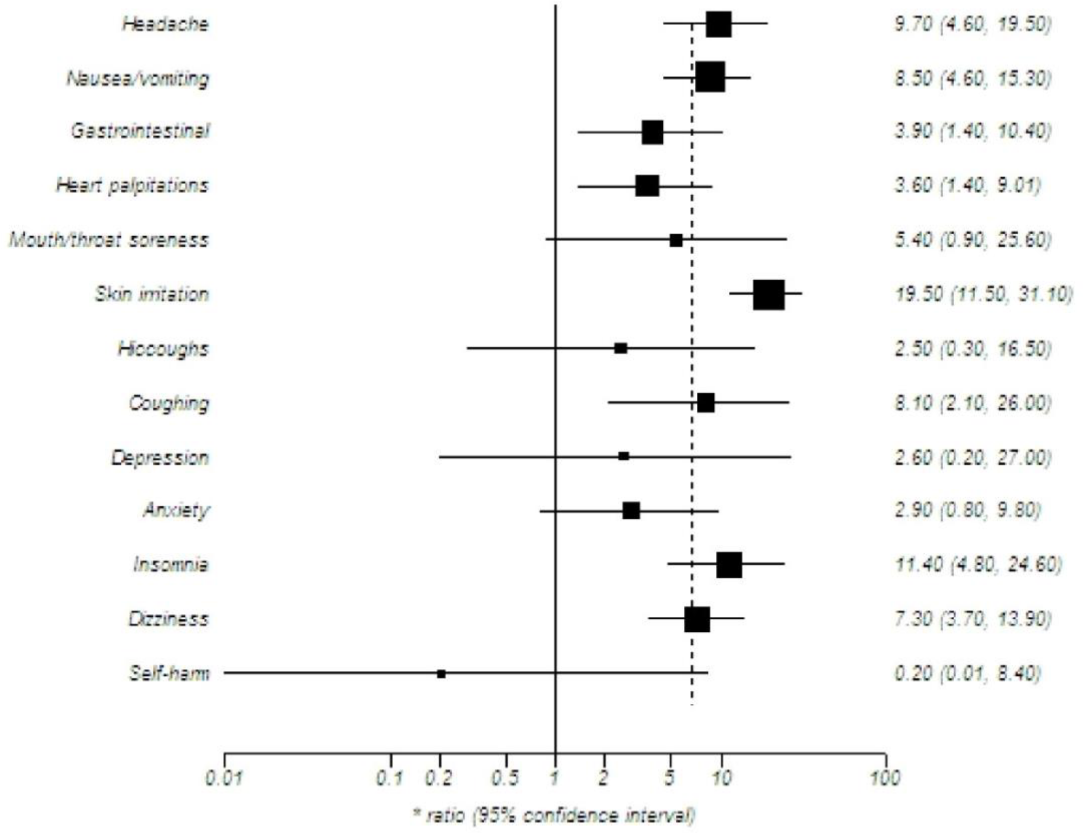

Bupropion (Zyban)

Mechanism of Action: Bupropion was originally developed as an anti-depressant and is still widely used for this purpose (Siu & Tyndale, 2007; Slemmer, Martin, & Damaj, 2000). Bupropion provides relief of withdrawal symptoms differently than nicotinic medications. The medications described above, Varenicline, patch and immediate release medications, bind to and stimulate nicotinic receptors. Bupropion on the other hand, is an atypical antidepressant with effects on dopamine and norepinephrine. During nicotine withdrawal there are decreased synaptic levels of dopamine (reward, satisfaction), norepinephrine (attention, alertness), and serotonin (sense of ease, peace) in neuronal synapses. Bupropion acts as a reuptake inhibitor increasing synaptic levels of dopamine and norepinephrine (but not Serotonin). As such, bupropion is thought to reduce feelings of anhedonia (inability to feel satisfaction/ reward) and concentration difficulties (alertness) that are major symptoms of withdrawal.

Rationale for Use: As a monotherapy, Bupropion vs. placebo shows OR = 1.85, which is roughly as effective as patch with OR = 1.9. Direct comparison of bupropion to patch shows no statistically significant difference. Bupropion in some studies produce a higher abstinence rate in smokers with high scores in depression (Stapleton et al., 2013).

Dosing: Treatment should begin while the patient is still smoking due to delayed treatment effect of 1 week. Patients should therefore set a "Quit Day" within the first 2 weeks of treatment with bupropion (GlaxoSmithKline, 2014). Total duration of treatment is variable, with some research protocols using 12 weeks and others as much as 6 months. Dosing is as follows (GlaxoSmithKline, 2014):
Days 1 -3: 150mg <u>once</u> daily (total dose 150mg/day)
Day 4: Remainder of treatment: 150mg <u>twice</u> daily with a minimum interval of 8 hours between doses (total dose = 300mg/day).
Further instructions for use: Allow at least 8 hours between doses and avoid taking prior to bedtime to minimize risk of insomnia (GlaxoSmithKline, 2014).

Contraindications: The major contraindicated for bupropion are individuals with seizure disorder as bupropion is known to lower the threshold for seizures. Those undergoing acute discontinuation of alcohol and sedatives such as benzodiazepines should also not be prescribed for the same reason of increased seizure risk. It is also contraindicated in individuals with a diagnosis of bulimia or anorexia nervosa (GlaxoSmithKline, 2014). Individuals using Wellbutrin should not be prescribed additional bupropion. Bupropion should also not be started in individuals who have been taking Monoamine Oxidase inhibitors (MAOi) in the previous 14 days. It should also be avoided in those with hepatic impairment, pregnant women and if <18 years old (GlaxoSmithKline, 2014).

Common Side effects: The most commonly reported adverse reactions of bupropion (incidence ≥5% and ≥1% more than placebo rate) are: sleep disturbances, dry mouth, rhinitis, dizziness, anxiety, nausea, constipation, and arthralgia (GlaxoSmithKline, 2014).
Insomnia: 30 - 40%
Dry mouth: 10%
Constipation, pruritis and pharyngitis: <10%

Approximately 7-12% of individuals who report these adverse effects discontinue using bupropion, although some studies have reported 31 - 33% of individuals on a 300mg dose of bupropion discontinuing medications due to side effects and 25% of those on 150mg discontinuing medication due to side effects (J. R. Hughes, Stead, Hartmann-Boyce, Cahill, & Lancaster, 2014; Richmond & Zwar, 2003). Allergic reactions such as pruritus, hives, angioedema and shortness of breath have been described at a rate of 1-3 per thousand (J. R. Hughes et al., 2014; Richmond & Zwar, 2003).

5.2.1. Pre-clinical experience of study drug

All medications used are FDA-approved for smoking cessation. Nicotine patch is provided over the counter without a prescription. Varenicline and Bupropion may be prescribed by primary care medical providers without clinical specialization. No drugs in this study are investigational and all have passed phase 1, 2 and 3 drug trials. Because only FDA-approved medications are used for this study, and all drugs are commonly used in the general population, drug development and safety data has not been provided. Links to FDA reports are provided below:

FDA data on Nicotine patch is provided at
http://www.accessdata.fda.gov/drugsatfda_docs/label/2015/020165Orig1s036lbl.pdf

FDA data on Bupropion (Zyban) is provided at:
http://www.fda.gov/safety/medwatch/safetyinformation/ucm176815.htm

FDA data on Varenicline (Chantix) is provided at:
http://www.fda.gov/drugs/drugsafety/ucm436494.htm and at http://www.fda.gov/drugs/drugsafety/ucm330367.htm

5.2.2. Clinical experience

All medications used are FDA-approved for smoking cessation. Nicotine patch is provided over the counter without a prescription. Varenicline and Bupropion may be prescribed by primary care medical providers without clinical specialization. No drugs in this study are investigational and all have passed phase 1, 2 and 3 drug trials. Because only FDA-approved medications are used for this study, and all drugs are commonly used in the general population, drug development and safety data has not been provided. Links to FDA reports are provided above.

5.3 Study Purpose/Rationale

Preliminary studies at Duke University have demonstrated a single method to provide targeted therapy to people that smoke through an adaptive pharmacotherapy algorithm ('Algorithm')(Christenhusz, Pieterse, Seydel, & van der Palen, 2007; Rose, 2011; Rose & Behm, 2013, 2014; Rose et al., 2009). Algorithm-based treatment results show two patterns of response to pre-quit medication treatment: (1) *Responders*, or those who show a 50% or more drop in cpd during the first 2 weeks of pre-quit treatment show higher abstinence rates overall with pre-quit medication alone, and show minimal or no benefit from adding Bupropion; and (2) *Non-Responders,* or people who do not decrease their rate of smoking substantially on pre-quit medications show a significant increase in abstinence rates with the addition of Bupropion. Subjects are assessed after 2 weeks of pre-quit treatment, and then non-responders are given bupropion in addition to initial treatment.

6 OBJECTIVES AND ENDPOINTS

	Objective	Endpoints	Analysis
**Primary**	To compare 30-day continuous biochemically confirmed abstinence at 12-weeks post-TQD in participants randomized to adaptive pharmacotherapy algorithm vs. standard-of-care treatment.	Biochemically confirmed 12-week post-TQD 30-day continuous abstinence.<br><br>This includes Carbon Monoxide Breath Testing < 7 ppm and single-item self-report of 30-day continuous abstinence.	See Section 13
**Secondary**	To compare 7-day point prevalent biochemically confirmed abstinence rates at 2- and 12-weeks post-TQD in participants randomized to adaptive pharmacotherapy algorithm vs. standard-of-care treatment.	Biochemically confirmed 2-week and 12-week post-TQD 7-day point prevalence abstinence.<br><br>This includes Carbon Monoxide Breath Testing < 7 ppm and a 7 day diary of cigarette use completed prior to in-person visits.	See Section 13
**Secondary**	To compare phone-based self-reported abstinence at multiple time points (1, 2, 6, 12, 26 and 52 weeks post-TQD) assessed by phone in participants randomized to adaptive pharmacotherapy algorithm vs. standard-of-care treatment.	Self-reported 7-day point abstinence rates via phone-based assessment at 24 hours post-TQD, 1-week post-TQD, 2-week post-TQD, 6-week post-TQD, 26-week post-TQD, and 52-week post-TQD.<br><br>This includes phone-based assessment of single-item question on smoking over the last 24 hours (for 24-hour and 1-week phone call assessment time points) and over the last 30 days for 26-week to 52-week time points.	See Section 13
**Secondary**	To compare smoking reduction (number of cpd) at 2- and 12-weeks post-TQD in participants randomized to adaptive pharmacotherapy algorithm vs. standard-of-care treatment.	Self-reported abstinence via diary correlated with biochemically-confirmed 2-week and 12-week post-TQD 7-day point prevalence abstinence.	See Section 13
**Secondary**	To compare smoking reduction (number of cpd) and on phone-based self-report at 1, 2, 6, 12, 26 and 52 weeks post-TQD in participants randomized to adaptive pharmacotherapy algorithm vs. standard-of-care treatment. Phone assessments will be compared at specific time points and also across multiple time points using multi-level modeling.	Self-reported abstinence rates via phone-based assessment at 24 hours post-TQD, 1-week post-TQD, 2-week post-TQD, 6-week post-TQD, 26-week post-TQD, and 52-week post-TQD.	See Section 13
**Exploratory**	To assess demographic variables (age, gender, race, education, and income) for an association with all abstinence outcomes and, if significant, will be used as covariates in analysis of all abstinence outcomes.	Self-reported demographic information assessed through surveys administered at the baseline visit.	See Section 13
**Exploratory**	To assess potential baseline (pre-intervention) predictors of abstinence including self-reported dependence, stress, anxiety, depression, alcohol and drug use, and self-efficacy. If a baseline variable is found to be associated with abstinence, it will be further assessed as a moderator of abstinence.	Self-reported baseline standardized measures on these psychological constructs assessed through surveys administered at the baseline visit.	See Section 13
**Exploratory**	To assess change in self-reported measures at baseline (pre-quit) to 2- and 12-weeks post-TQD on repeated standardized measures of nicotine dependence, stress, anxiety, depression, and self-efficacy. If a	Self-reported baseline and also 2 and 12 week post quit standardized surveys on these psychological measures assessed through in-person surveys	See Section 13

	measure is found to change significantly from baseline to a post quit time point, it will be assessed as possible mediator of abstinence outcomes.		
**Exploratory**	To compare baseline scores on anxiety, depression, and stress for their effect on allocation to Responder or Non-Responder in the adaptive and standard treatment.	Self-reported baseline standardized measures	See Section 13
**Exploratory**	To compare phone-assessed non-standardized single-item questions on urges, withdrawal, confidence and motivation at 1, 2, 6, 12, 26 and 52 weeks post-TQD. If a measure is found to change significantly from baseline to a post quit time point, it will be assessed as possible mediator of abstinence outcomes. Phone assessments will be compared at specific time points and also across multiple time points using multi-level modeling.	Self-reported response via phone-based assessment at 48 hours post-TQD, 1-week post-TQD, 2-week post-TQD, 6-week post-TQD, 26-week post-TQD, and 52-week post-TQD on individual non-standardized questions on each of these psychological constructs.	See Section 13
**Exploratory**	To conduct a cost-benefit analyses on allocation to adaptive pharmacotherapy algorithm vs. standard-of-care treatment from the perspective of multiple stake holders: smokers, healthcare systems (clinics/hospitals), insurance companies, and employers.	Biochemically confirmed abstinence measures as well as phone measures, demographic measures and financial questions and work questions (see Appendix 3 and 12)	See Section 13
**Feasibility**	Demographics of patients at DSCP and Duke CSC	Demographic variables assessed	See Section 13
**Feasibility**	Medications used	Medication selection at randomization	See Section 13
**Feasibility**	Attendance at DSCP appointments	Attendance recorded in EMR	See Section 13
**Feasibility**	Referral routes to DSCP	Referral clinic/route recorded	See Section 13
**Feasibility**	Medication side effects	Side effects queried during in-person and phone call assessments	See Section 13
**Feasibility**	Medication adherence	Medication use as reported via daily diary 7 days prior to in-person visits.	See Section 13

7 INVESTIGATIONAL PLAN

7.1 Study Design

This study is a double-blinded, randomized-controlled trial. Participants will be randomized to Varenicline- or Nicotine Patch-based adaptive pharmacotherapy algorithm or to standard treatment with Varenicline or the Nicotine patch.

7.1.1. Dose Modification

Dosing of medications will follow FDA recommendations. If a participant experiences a side effect or symptom that may be ameliorated by dose adjustment (e.g. nausea while using the nicotine patch), the study physician or Physician Assistant will make these adjustments. The decisions to make the adjustments will be based on clinical judgment.

7.1.2. Safety Considerations

All study participants will be provided with a list of possible common adverse events related to all of the medications used within the consent form. They will be asked to contact the CTA by phone if they experience side effects. They will also be given contact information for the study Medical Supervisor (Dr. Davis). All information on side effects will be directed to the study Physician or Physician Assistant. Additional monitoring will be provided at every phone call by the CTA. The participants are asked about possible side effects just after starting medications, before, on and just after the quit day and at other time points.

7.1.3. Missed Doses

CTA will ask about the use of medications and will enter information about missed doses into their report. At each visit, the CTA will request that the participant bring all of their medication. The CTA will count the remaining pills and record this for medication compliance monitoring.

7.1.4. Concomitant Medications/Therapies

Participants enrolled in the study will continue on the medications that they are taking at the time they start the study. They will be asked not to take additional tobacco use treatment medications during the study. If they start new medications during the study, they will be asked to report this to the CTA.

7.1.5. Study Drug Blinding

Subjects are blinded to their active medications. Study personnel who are in contact with participants, including the CTA, study clinician, and PI, are blinded to active medication.

7.1.6. Randomization

Randomization will occur through an open source randomization program “Research Randomizer” developed for randomization of subjects in psychology and medication trials. This can be found at https://www.randomizer.org/. To simulate clinical treatment, the study design provides for subject choice into one of two arms: Varenicline-based treatment (n=150) or Nicotine Patch-based treatment (n=150). Once the initial treatment arm is chosen, subjects will be randomized to Varenicline adaptive pharmacotherapy (n=75) vs. Varenicline alone (n=75), or similarly nicotine patch adaptive treatment (n=75) vs. nicotine patch alone (n=75).

7.2 Rationale for Selection of Dose, Regimen, and Treatment Duration

All dosing schedules used are based on FDA-approved dosing for these FDA-approved medications - but with minor modifications to fit study design requirements. For example:

Varenicline: Varenicline for control group is dosed exactly as normally prescribed but is continued for 12 weeks instead of 11 weeks post quit. In study group subjects, Varenicline is also used exactly as normally prescribed but is started 4 weeks prior to TQD instead of 1 week prior.

Bupropion: Bupropion is dosed exactly as is described for smoking cessation (started 1 week prior to quit day, continued for 12 weeks).
Nicotine Patch: Nicotine patch is dosed according to patient's use of nicotine – smokers who use 10 cpd or fewer will be on 14 mg patch, those who smoke 11-20 cpd will use a 21 mg patch and those who smoke over 20 cpd will be on a 42 mg patch. This parallels findings in our trial on adaptive treatments using nicotine patch in which participants were randomized to pre-quit nicotine patch of 21 mg vs. 42 mg with the finding that high use smokers showed higher abstinence (with allocation arms combined) using 42 mg Nicotine Patch, whereas low-use smokers showed higher abstinence (with allocation arms combined) using a 21 mg Nicotine Patch. In a follow-up study, participants were given a nicotine patch of 42 mg for CO >30 and 21 mg patch for CO ≤ 30. Post-hoc regression analysis showed that a CO level of 30 was most highly correlated with 23 cigs/day (Rose & Behm, 2013).

7.3 Rationale for Correlative Studies

An observational study is being conducted on all patients of the DSCP, as well as a phone-based intervention study. Enrollment in either of these studies will exclude a participant from enrolling in this trial.

A qualitative study is being conducted on shared-decision making in lung cancer screening that may include some overlap of participants as well. However, this study is purely qualitative and includes no pharmaceutical or behavioral interventions so should not interfere with the present study being conducted.

7.4 Definition of Evaluable Subjects, On Study, and End of Study

Participants who enroll in the study will be "evaluable." De-identified data from subjects who sign consent for screening will also be used for creation of the Consort Diagram – to show which individuals were excluded or did not sign consent vs. those who were enrolled. Data from subjects who withdraw from the study will still be considered evaluable.

7.5 Early Study Termination

This study can be terminated at any time for any reason by the PI-sponsor – the National Institutes on Drug Abuse. If this occurs, all subjects on study will be notified as soon as possible. Additional procedures and/or follow up should occur in accordance with Section 10.6, which describes procedures and process for prematurely withdrawn patients.

8 STUDY DRUG

8.1 Names, Classification, and Mechanism of Action

Varenicline Tartrate
Alternate names: Varenicline, Chantix
Type of Study drug class: smoking cessation medication
Mechanism of action: Acetylcholine-Nicotinic receptor partial agonist/antagonist

Bupropion Hydrochloride – sustained release
Alternate names: Bupropion SR, Zyban, Wellbutrin.
Type of Study drug class: Antidepressant, appetite suppressant, smoking cessation medication
Mechanism of action: inhibits reuptake of synaptic dopamine and norepinephrine.

Nicotine patch
Alternate names: Nicoderm, transdermal nicotine patch
Type of Study drug class: smoking cessation medication
Mechanism of action: Acetylcholine-Nicotinic receptor agonist

8.2 Packaging and Labeling

All medications will be labeled according to Investigational Chemotherapy Service (ICS) Standard Operating Procedures to include at least: drug name/strength, quantity, lot number, and expiration or retest date. Placebo medications will be provided in non-descript packaging, similarly to non-placebo medications.

8.3 Supply, Receipt, and Storage

Pfizer will provide Varenicline and placebo Varenicline; nicotine patches, bupropion and placebo bupropion will be provided by Central Compounding Pharmacy Incorporated. CVS brand clear adhesive bandages will be purchased to serve as placebo nicotine patches. For individuals who do not have an adhesive allergy, but instead have other forms of skin sensitivity such as discoloration, redness, tenderness (which can be related to the adhesive used in specific brands of patch), an alternate placebo or active patch will be purchased. This will provide an alternative for participants who express concerns about these types of issues but would like to continue using nicotine patch. ICS will be responsible for managing the receipt and storage of all pharmaceuticals and placebo medications.

8.4 Dispensing and Preparation

Pharmaceuticals will be prepared and dispensed according to ICS Standard Operating Procedures. Study staff will collect the participant's pharmaceuticals from the ICS once the participant has arrived at the clinic and dispense them during the Study Visit.

8.5 Compliance and Accountability

Participants will be asked to return un-used medications at each visit. There are 4 study assessment visits at which medication management (Unused Medication Count and Dispensing Medications) will occur: Enrollment visit, Medication Response Visit, 2-week post quit visit, 12-week post quit visit:

Enrollment Visit: Provide 3 weeks of Medications
Medication Response Visit: (2-weeks after Enrollment visit): Provide 4 weeks of Medications (through quit day and through 2-week post quit visit) Unused medication count)
2-week post Quit Visit: (4 weeks after Medication Visit): Provide 9 weeks of Medications (through 12-week post quit visit) (Unused medication count)
12-week post quit visit: (10 weeks after 2-week post quit visit) (Unused medication count)

During each Unused Medication Count, the participant will bring unused medications to the study assessment visit. The CTA will then count these medications and record the outcome for an assessment of medication adherence. This data will also be recorded at ICS. In addition, ICS will collect all used medication bottles.

8.6 Disposal and Destruction

ICS will be responsible for destruction of unused medications and will follow standard research pharmacy procedures for this.

9 SUBJECT ELIGIBILITY

Patients who consent to participate in the study will be screened for the following inclusion/exclusion criteria.

9.1 Inclusion Criteria (assessed by research staff and/or study medical provider)

1. Age 18 years or older
2. Actively smoking 5 or more cigarettes per day for at least one year
3. Have a median CO of at least 7 ppm (on carbon monoxide breath test)
4. Willing to set a quit date within 6 weeks
5. Willingness to take Varenicline OR nicotine patch (patient choice) and Bupropion
6. Access to a telephone

9.2 Exclusion Criteria (assessed by research staff and/or study medical provider)

1. 5 or above for women OR 6 or above for men on the Alcohol Use Disorders Identification Test (AUDIT-C) are considered a "positive" screen and will require additional clinician assessment to determine if an alcohol use disorder is present.
2. Illicit drug use within the last month
3. 3 or above on Patient Health Questionnaire (PHQ-2) Depression Scale or >0 in PHQ-9 question 9
4. Daily use of a second form of tobacco or nicotine or smoking a product other than combustible cigarettes (e.g. e-cigarettes, cigars, chewing tobacco, snuff, hemp)
5. Current use of a smoking cessation medication (e.g. nicotine replacement, Varenicline, Bupropion)

6. Report of pregnancy, attempting to get pregnant, or actively breast feeding or positive urine pregnancy test (only given to females with child bearing potential)

9.3 Exclusion Criteria (assessed study medical provider)

7. Medical contraindication to the use of Varenicline or Nicotine Patch, or Bupropion (including seizure disorder)
8. Brain abnormality that would increase the patients risk for a seizure (including but not limited to a stroke or brain tumor)
9. Bulimia or anorexia
10. Currently undergoing discontinuation of alcohol or sedative (including benzodiazepines, barbiturates, or antiepileptic drugs)
11. Use of MAO-inhibitor (currently or in the last 14 days)
12. Taking Linezolid or intravenous methylene blue.
13. Significant adverse reaction to bupropion in the past
14. Symptomatic cognitive or emotive disorder such as untreated schizophrenia, severe untreated depression, anxiety.
15. Uncontrolled hypertension (systolic BP >160 mm Hg, diastolic BP >100 mm Hg) or hypotension
16. Chest pain in the last month (unless history indicates a non-cardiac source) or myocardial infarction in the past year
17. Unstable irregular heart rhythm
18. Symptomatic cardiac disorder
19. Moderate renal impairment or mild hepatic impairment (study provider discretion)
20. Current symptomatic lung disorder/disease (including but not limited to COPD, emphysema, and asthma) that requires oxygen
21. Migraine headache occurring more than once per week
22. Unexplained fainting spell within the last month
23. Uncontrolled diabetes (blood sugars >200 more than once per week, suffering from complications or on insulin)
24. Current cancer or treatment of cancer in the past 6 months (except basal or squamous cell skin cancer)
25. History of Tuberculosis or positive PPD
26. Suicidal ideating within the past 12 months or attempted suicide within the last 10 years.
27. Psychiatric hospitalization in the past 12 months
28. Red dye allergy
29. Any other major medical conditions (study provider discretion)

10 TRIAL PROCEDURES AND ASSESSMENTS

Relationship of this study to the Duke Translational Smoking Cessation Program (TSCP) and Duke Center for Smoking Cessation (CSC)

Study recruitment will take place among a clinical population via three routes - the TSCP Clinics, Duke Primary Care (DPC) Clinics, and the Center for Smoking Cessation (CSC).

Duke Translational Smoking Cessation Program (TSCP): Most patients in this study will be recruited through the Duke TSCP clinics – a network of six clinics throughout the Duke University Health System (DUHS) that receive roughly 250 patient referrals per month. The TSCP is a growing program expanding to serve patients across Duke Health. Dr. James Davis, the PI for this study, is Director of the TSCP and oversees all TSCP personnel and processes. Each TSCP Clinic site contains a provider trained in tobacco cessation treatment (typically an APP (Advanced Practice Provider)) who performs a thorough evaluation, conducts physical exams and prescribes medication and a Medical Assistant or Staff Assistant who assists with data collection. The TSCP is designed with a research infrastructure in which data is collected in a standardized format and research staff who recruit patients are present on site for studies. The TSCP contains centralized operations including a clinic coordinator who calls patients for scheduling and follow up. Every patient who is scheduled for an appointment at a clinic within the TSCP will be screened for research study eligibility. If a patient could potentially be eligible for a research study, a note will be made in their medical chart. Research staff will then attempt to reach out to them to see if they are interested in participating in a research study (via MyChart message and then via phone). If the patient is interested in the study and meets the inclusion criteria, the patient will be scheduled to meet research staff at the TSCP clinic or the CSC for a screening visit. If the potential participant is not interested in a research study or not eligible, a note will be made in their medical record and they will be encouraged to keep their regularly scheduled clinic visit.

Duke Primary Care (DPC): The study will also recruit participants through the Duke Primary Care (DPC) Clinics. Authorization has been provided by the DPC Research Consortium, which will provide access to smokers who have consented to be contacted for research. The Research Consortium manages multiple studies to avoid conflict between studies and will provide access to three DPC Clinics. DPC includes a network of 40 primary care clinics throughout Duke University Health System. Patients who attend visits with DPC medical providers at within Duke Primary Care are screened to see if they are willing to be contacted to find out about available research. DPC patients who are identified within the Electronic Health Record as being “current smokers” will be available for contact for our study. Using a regularly generated report, research staff will use participant MRN to look at their medical record to screen for eligibility (name, mailing address, email address, phone number, age, smoking history, allergies, emergency department visits for substance use, medications, and medical diagnoses). Ineligible participants will not be contacted. If eligible, research study staff will contact the eligible participant via phone and MyChart message to describe the study. MyChart messages will include information on opting out (center phone number). Any voicemails left will be non-descript and only including brief information that they may be eligible for a research study at Duke and a phone number to reach back out (no information provided on smoking status). If participants have previously opted-out of research (as noted in their medical record), they will not be contacted. If they are interested and meet inclusion criteria, the research staff will schedule them for a screening visit at the Duke Center for Smoking Cessation (CSC) (central study site).

Other Research Studies: Potential subjects will be recruited through pre-screen failures from other studies at the CSC. There are currently several studies recruiting smokers from communities in and around Durham, North Carolina through newspaper flyers, internet and TV advertisements, and word-of-mouth. If potential subjects fail to meet the pre-screening study requirements for these studies (Pro: 00074741, Pro: 00042277) at the CSC, but are still interested in research, they will be routed to a CTA to be pre-screened for this study over the phone. If potential subjects meet the pre-screening study requirements and are still interested in participation, they will attend a physical screening session at the Duke CSC, located at 2424 Erwin Road, Suite 201, Durham, NC 27705. At the screening on-site visit, a CTA will meet with the patient in a private area of the CSC (consent room) and provide the patient with the Study Consent Form for review.

Advertisements: Subjects will be recruited through internet-based ads on free websites (for example, Craigslist). The ad will feature a phone number and contact email for potential participants and can be found in the Recruitment section. A physical copy of this ad may also be produced to be used as a flyer advertising the study in Durham and the surrounding areas.

Consent: Interested individuals will be provided with the Study Consent Form (CF), which provides a description of the study. The CTA will review the consent form with the potential participant, read key sections out loud, clarify terms as needed, and answer questions. Ability to read and understand English will be assessed at this time. If unable, the consent process will be discontinued. After reviewing the CF, if the interested individual is interested in participation in the study, they will they will be asked to sign consent in order to continue screening (completion of exclusion criteria and pregnancy testing). This will include assessment of data collected in EPIC specifically for the Duke Smoking Cessation Program Initial Clinic Visit. The study medical staff (PA or MD) will have access to the potential participant's EPIC record in order to review the Duke Smoking Cessation Program Initial Clinic visit for participant eligibility as well as other pertinent elements of the patient's medical record to ensure their safe inclusion in the study. Individuals who pass Inclusion/Exclusion Criteria will be required to take a urine pregnancy test if they are of child-bearing potential, as defined by the Duke IRB.

SCHEDULE OF ACTIVITIES							
	**Screening Phase**	**Treatment Phase**			**End of Treatment**	**Phone Follow up Period**	**End of Study**
	**Screening Visit**	**Enrollment Visit**	**Medication Response Visit**	**2-week post Quit Visit**	**12-week Post Quit Visit (Discontinue Medications)**	**Calls 1-9**	**52-week post quit phone call**
**Assessments/Observations**							
Assessment in-person	X	X	X	X	X		
Obtain Informed Consent	X						
Assessment of Inclusion/Exclusion Criteria. Urine pregnancy test	X						
Vitals and CO breath test	X	X	X	X	X		
Psychometric measures	X			X	X		
Dispense Medications		X	X	X			
Assess Medications			X	X	X		
Phone Call Assessments	X	X	X	X	X	X	X
Assess Adverse Events		X*	X	X	X	X	X
Payment		X	X	X	X		X

*The Adverse Events questionnaire will be administered at the Enrollment visit in order to obtain baseline data on a patient's symptoms to better understand if the development of any SE is related to study drugs.

10.1 Screening Examination

The screening examination will take place on the day of the screening visit. Subject data to be collected at the Screening Examination includes the following baseline measures: contact information questionnaire, demographics questionnaire, smoking-related medical history questionnaire, TSCP smoking history questionnaire, recent smoking history questionnaire, medication use and intolerance questionnaire, financial questionnaire, work break questionnaire, cost of smoking questionnaire, general health questionnaire, cancer questionnaire, self-efficacy questions, Fagerstrom Test for Nicotine Dependence (FTND), Mood and Physical Symptoms Scale (MPSS), Patient Health Questionnaire (PHQ-2 as well as PHQ-9 question 9), Generalized Anxiety Disorder-2 (GAD-2), Alcohol Use Disorders Identification Test (AUDIT-C), and the Perceived Stress Test (PSS-4). The subject will also complete a urine pregnancy test if female and of child-bearing potential.

The urine pregnancy test will be conducted by the CTA or other study staff with the appropriate training. Most information except the urine pregnancy test results will be documented in the EMR because these assessments are all part of standard clinical practice within the TSCP. If the screening visit occurs at the CSC, outside of the normal TSCP clinic, all information will be collected on paper forms or in REDCap. If the screening visit occurs at the TSCP during a routine clinic visit, the study Physician or Physician Assistant will review the visit documentation to assess eligibility. In the event of a screen failure, documentation of this will be noted in eResearch. Participants that fail the screening process will receive follow-up from the study physician to ensure the participant has been connected to appropriate clinical care.

Enrollment/Randomization Visit: If an individual meets the criteria to be enrolled in the study, they will be asked to come to the Enrollment/Randomization Visit. At this visit, the individual will be enrolled (given a study ID) and randomized to adaptive vs. non-adaptive treatment. The CTA will notify the research pharmacy of the enrollment and randomization status and will pick up medications/placebos for the participant then give these to the participant with a description of how to use them. The participant will then be scheduled for follow up visits in 2 weeks (Medication Response Visit) and 2- and 12-week post quit. They will also be entered into the phone-based follow up system (see Phone-based Assessment Time Table below). At the point of Enrollment, all payment for TSCP services will stop, and participants receive free treatment, medications etc. through the study.

10.2 Treatment Period

The following is an overview of active treatments and then active and placebo treatments for participants allocated to adaptive therapy and standard therapy groups:

Figure 2. Adaptive Pharamcotherapy Protocol (n=150)

**VARENICLINE-BASED TREATMENT**<br>Participant choses Varenicline-based treatment and is then randomized to Adaptive Treatment arm (N=75)	**NICOTINE PATCH-BASED TREATMENT**<br>Participant choses Nicotine patch-based treatment and is then randomized to Adaptive Treatment arm (N=75)
**VARENICLINE NON-RESPONDER**<br>4 weeks prior to TQD: Start Varenicline<br>2 weeks prior to TQD, DOES NOT reduce cigs/day by > 50%<br>1 week prior to TQD: Start Bupropion<br>Use Varenicline + Bupropion to 12 weeks post TQD	**NICOTINE PATCH NON-RESPONDER**<br>4 weeks prior to TQD: Start Nicotine Patch<br>2 weeks prior to TQD, DOES NOT reduce cigs/day by > 50%<br>1 week prior to TQD: Start Bupropion<br>Use Nicotine Patch + Bupropion to 12 weeks post TQD
**VARENICLINE RESPONDER**<br>4 weeks pre-TQD: Start Varenicline<br>2 weeks prior to TQD - DOES reduce cigs/day by > 50%<br>1 week prior to TQD: Start Placebo Bupropion<br>Use Varenicline + Placebo Bupropion to 12 weeks post TQD	**NICOTINE PATCH RESPONDER**<br>4 weeks pre-TQD: Start Nicotine Patch<br>2 weeks prior to TQD - DOES reduce cigs/day by > 50%<br>1 week prior to TQD: Start Placebo Bupropion<br>Use Nicotine Patch + Placebo Bupropion to 12 weeks post TQD

Standard Treatment Regimens: Participants who chose either Varenicline or Patch-based treatment but who are randomized to standard treatment will be given Varenicline or Nicotine Patch with what is now considered standard-of-care treatment. Varenicline Standard Treatment arm will start Varenicline 1 week prior to the TQD and continue for 12 weeks post TQD. Nicotine Patch Standard Treatment arm will start nicotine patch on the TQD and continue for 12 weeks post TQD. Placebo medications are used to match Adaptive Treatment Protocols and shown in Figure 3.

Figure 3. STANDARD PHARMACOTHERAPY PROTOCOLS (n=150)

**VARENICLINE-BASED TREATMENT**<br>Participant choses Varenicline-based treatment and is then randomized to Standard Treatment arm (N=75)	**NICOTINE PATCH-BASED TREATMENT**<br>Participant choses Nicotine patch-based treatment and is then randomized to Standard Treatment arm (N=75)
**VARENICLINE STANDARD TREATMENT**<br>4 weeks pre-TQD: Start Placebo Varenicline<br>1 week prior to TQD – Switch to Active Varenicline<br>1 week prior to TQD: Start Placebo Bupropion<br>Use Varenicline + Placebo Bupropion to 12 weeks post TQD	**NICOTINE PATCH STANDARD TREATMENT**<br>4 weeks pre-TQD: Start Placebo Nicotine Patch<br>TQD - start Nicotine Patch<br>1 week prior to TQD: Start Placebo Bupropion<br>Use Nicotine Patch + Placebo Bupropion to 12 weeks post TQD

Medication Dosing for Each Group:

Varenicline Adaptive Treatment Arm (n=75)

Participants who chose Varenicline-based treatment and are then randomized to the Varenicline Adaptive Treatment Arm will receive the following:

4 weeks prior to TQD: Begin Varenicline with this schedule:

Day 1-3: 0.5 mg q AM for 3 days (with placebo Varenicline in the evening)
Day 4-7: 0.5 mg BID
Day 8 to end of treatment: 1 mg BID

This group will remain on 1 mg BID Varenicline x 3 weeks pre-TQD, then 12 weeks post TQD.

At the 2 week pre-TQD visit, this group will be assessed for “Response” to Pre-quit Varenicline:

a. Varenicline Non-Responders: Participants who do NOT show > 50% reduction in cigarettes-per-day from baseline will be continued on Varenicline, and Bupropion will be added as a "rescue medication."
Bupropion will be added to their regimen 1 week prior to the TQD with this schedule:
Day 1-3: 150 mg q AM
Day 4 to end of treatment: 150 mg BID
Bupropion will be continued for 12 weeks post TQD.

b. Varenicline Responders: Participants who show > 50% reduction in cigarettes per day from baseline will be continued on Varenicline as their only active medication.
1 week prior to the TQD, Placebo Bupropion will be added with this schedule:
Day 1-3: 1 placebo pill q AM
Day 4 to end of treatment: 1 placebo pill BID
Placebo Bupropion will be continued for 12 weeks post TQD.

Varenicline Standard Treatment Arm (n=75)

Participants who chose Varenicline-based treatment and are then randomized to the Varenicline Standard Treatment Arm will receive the following:
4 weeks prior to TQD: start Placebo-Varenicline 1 pill BID
1 week prior to TQD: stop placebo Varenicline and start real Varenicline on the following start up schedule:
Day 1-3: 0.5 mg q AM for 3 days (with placebo Varenicline in the evening)
Day 4-7: 0.5 mg BID
Day 8 to end of treatment: 1 mg BID
This group will remain on Varenicline 1 mg BID x 12 weeks post TQD.
1 week prior to the TQD: Placebo Bupropion will be added:
Day 1-3: 1 pill q AM
Day 4 to end of treatment: 1 pill BID
Placebo Bupropion will be continued for 12 weeks post TQD.

Nicotine Patch Adaptive Treatment Arm (n=75)

Participants who chose Nicotine Patch-based treatment and are then randomized to the Nicotine Patch Adaptive Treatment Arm will receive the following:
4 weeks prior to TQD: start on Nicotine Patch with the following dosing:
14 mg/day for participants smoking 5-10 cigarettes per day
21 mg/day for participants smoking 11-20 cigarettes per day
42 mg/day for participants smoking 20 or more cigarettes per day
This group will remain on this treatment for 4 weeks pre-TQD, then 12 weeks post TQD.
At the 2 week pre-TQD visit, this group will be assessed for "Response" to pre-quit Nicotine Patch:

a. Nicotine Patch Non-Responders: Participants who do NOT show > 50% reduction in cigarettes-per-day from baseline will be continued on Nicotine Patch, and Bupropion will be added as a "rescue medication".
1 week prior to the TQD: Bupropion will be added to their regimen with this schedule:
Day 1-3: 150 mg q AM
Day 4 to end of treatment: 150 mg BID
Bupropion will be continued for 12 weeks post TQD.

b. Nicotine Patch Responders: Participants who show > 50% reduction in cigarettes-per-day from baseline will be continued on Nicotine Patch as their only active medication.
1 week prior to the TQD: Placebo Bupropion will be added
Day 1-3: 1 pill q AM
Day 4 to end of treatment: 1 pill BID
Placebo Bupropion will be continued for 12 weeks post TQD.

Nicotine Patch Standard Treatment Arm (n=75)

Participants who chose Nicotine Patch-based treatment and are then randomized to the Nicotine Patch Standard Treatment will receive the following:
4 weeks prior to TQD they will be started on placebo Nicotine Patch once per day.

On the TQD, placebo Nicotine Patch will be stopped and real Nicotine patch will be started with this dosing:

14 mg/day for participants smoking 5-10 cigarettes-per-day

21 mg/day for participants smoking 11 or more cigarettes-per-day

This group will remain on Nicotine Patch once per day x 12 weeks post TQD.

1 week prior to the TQD, Placebo Bupropion will be added:

Day 1-3: 1 pill q AM

Day 4 to end of treatment: 1 pill BID

Placebo Bupropion will be continued for 12 weeks post TQD

10.3 End of Treatment

At the end of treatment (12-week post-quit study visit) study staff will administer questionnaires (detailed further in section 10.7) and a program evaluation questionnaire. Participants will return all unused medications and empty pill bottles.

10.4 Follow-up Period

Following the End of Treatment, participants will receive two follow-up phone assessments (detailed further in section 10.7). These phone assessments will occur at 26-weeks post-TQD and 52-weeks post-TQD. If participants are not currently abstinent from tobacco at the End of Treatment, a referral to clinical resources within the TSCP will be offered.

Follow up Study Assessment Visits: At each assessment visit, there will be a standardized evaluation. The screening will be provided through the Duke Smoking Cessation Program for clinical purposes. Later assessments (Enrollment, Medication Response, 2- and 12-week post quit) will be provided by the CTA. During these visits there will be multiple self-report assessments (see below) as well as CO testing. Finally each participant will receive a total of 10 phone calls out to 1 year post quit attempt. Biochemically confirmed 30-day continuous abstinence at twelve weeks post-Target Quit Day is the primary endpoint to determine abstinence. Other endpoints, including biochemically confirmed 7-day point prevalent abstinence at two and twelve weeks post-TQD and self-reported abstinence rates via phone-based assessment up to 52-week post-TQD, will also assess abstinence as secondary measures.

Phone-based Assessment Time Table: The phone assessments function to collect data on study participants but also to check on difficulties with medications, medication side effects and to remind participants of upcoming visits. The timing is as follows:

1	1 day after the Enrollment Visit
2	1 day before the Medication Response Visit
3	1 day pre-TQD
4	1 day post-TQD
5	1-week post-TQD visit
6	1 day before the 2-week post-TQD visit
7	6-week post-TQD time point
8	1 day before the 12 week post-TQD visit
9	26 week post-TQD time point
10	52 week post-TQD time point

10.5 End of Study

Study participation will end with each participant after the 52-week post TQD phone assessment. If participants cannot be reached by phone, they will be contacted by additional means including email or other contact methods listed. If they are still not able to be contacted, they will be listed as “lost to follow up” for purposes of analysis of results (see management of missing data in analysis). If participants are not abstinent from tobacco at the End of Treatment, a referral to clinical resources within the TSCP will be offered. After all participants have completed the study, study data collection period will be considered over and the team will begin analysis of results. Analysis may continue for up to 5 years. This is necessary because of multiple sub-analyses that may be performed on the large quantity of data generated from this trial. After this 5-year period, it is expected that the trial will lock the data.

10.6 Early Withdrawal of Subject(s)

10.6.1. Criteria for Early Withdrawal

Subjects may voluntarily withdraw from the study at any time. The PI may also withdraw a subject from the study at any time based on his/her discretion. Reasons for PI-initiated withdrawal may include, but is not limited to the following:

- Adverse events
- Abnormal test procedure results
- Protocol deviation
- Administrative issues
- Disease progression
- Pregnancy

10.6.2. Follow-up Requirements for Early Withdrawal

If a participant has cause for premature withdrawal, or if the PI decides that the participant should withdraw, he or she will be asked to attend a Premature Withdrawal Visit. If this occurs before the 12-week post quit visit (final treatment visit), the Premature Withdrawal Visit will essentially mirror the 12-week post quit visit (if the subject is able). If the premature Withdrawal occurs after the 12-week visit, then the patient will simply be called on the phone for a final phone call assessment then withdrawn with payment for the encounters he/she was able to complete.

10.6.3. Replacement of Early Withdrawal(s)

Sample size estimation from this trial is all based on intent-to-treat data and attrition is expected and included within the calculation of abstinence rates for all outcomes. As such, there will be no replacement for premature withdrawal of subjects or subjects lost to follow up.

10.7 Study Assessments

10.7.1. Medical History

Medical history assessments will be completed by each subject as part of standard clinical practice. A Clinical Trials Assistant or other Key Personnel will review this as part of the screening process. Appendix 1 provides an overview of medical history information to be collected and is considered a baseline measure.

10.7.2. Physical Exam

A physical exam will be conducted as part of standard clinical practice by the Clinical Tobacco Treatment Specialist (TTS medical provider) at the initial visit to the TSCP or Duke CSC. Exam findings however, will not be used to exclude patients from participation in the study. The study only uses medications that are FDA approved for smoking cessation.

Subject-Rated Non-Standardized Measures

At each clinic visit, subjects will complete a number of assessments, some standardized and others non-standardized. All of these measures are completed by the subject as part of standard clinical practice. If the subject consents to research, the previously completed information will be recorded as Baseline measures. A Clinical Trials Assistant or other Key Personnel will review these as part of the screening process.

At the Initial visit to the TSCP or Duke CSC (baseline visit), the subject will complete the Smoking History questionnaire (Appendix 2). This non-standardized questionnaire provides information on the number of pack years, additional tobacco product use, and other drug use.

A subject's financial and insurance status is also assessed using the Financial Questions (Appendix 3). Cost of Smoking (Appendix 4) ascertains the amount the participant pays for cigarettes.

Medication Intolerance Questions (Appendix 5) determines if a participant's intolerance to a tobacco treatment medication or other contraindications to these medications (e.g. pregnancy) may exclude them from the study. Health and Surgery (Appendix 6) and Cancer Questions (Appendix 7) also measure if a participant should be excluded from the study for current symptomatic medical conditions. Race and ethnicity, are assessed in Demographics (Appendix 8).

10.7.3. Subject-Rated Standardized Measures

A participant's alcohol use is assessed at Baseline using the AUDIT-C (Appendix 9). A score of 3 or higher (if female) or 4 or higher (if male) is considered a "positive" screen, and will require additional assessment from the study clinician to determine if the participant can be included in the study..

10.7.4. Subject-Rated Non-Standardized Measures to Be Collected at All Clinic Visits

Thirty- and 7-day point prevalence are assessed in Recent Smoking (Appendix 11), as well as compliance with prescribed medications and reasons for non-compliance, if applicable.
A participant's employment status is assessed by Work Questions (Appendix 12); this measure also allows the study staff to calculate the amount of time spent during smoke breaks during the work day.
A participant's self-efficacy and motivation to stop smoking are assessed with Predictor Questions (Appendix 13).

10.7.5. Subject-Rated Standardized Measures to Be Collected at All Clinic Visits

A number of standardized measures are collected at each clinic visit. All references are listed in each respective appendix. A participant's level of physical nicotine dependence is assessed using the FTND (Appendix 14).
A participant's current withdrawal symptoms from cigarettes is assessed in the MPSS-2 (Appendix 15). Depression is assessed using the PHQ-2 (Appendix 16), anxiety is assessed using the GAD-2 (Appendix 17) and stress is assessed using the PSS-4 (Appendix 18).
Finally, participants will be given a 7 day daily diary to complete prior to in-person study visits. This diary will ask about the number of cigarettes smoked per day and daily medication adherence. Participants will be asked to complete this prior to their visit. If they have not completed the diary prior to the visit, the RA will use the time-line follow-back approach to complete the 7 day diary with the participant. This form can be found in Appendix 20.

10.7.6. Subject-Rated Measures to Be Collected through Phone Assessment

Participants will be followed up with by phone to answer questions about the following:

- Abstinence status
- Average number of cpd over the past 24 hours, 7 days, and 30 days
- Medications they are taking and side effects they are experiencing, if any
- Stress level
- Experience of urges
- Confidence in their ability to become tobacco free

These questions are also listed in Appendix 19

11 SAFETY MONITORING AND REPORTING

The PI is responsible for the identification and documentation of adverse events and serious adverse events, as defined below. At each study visit, the PI or designee must assess, through non-suggestive inquiries of the subject or evaluation of study assessments, whether an AE or SAE has occurred.

The DCI Safety Surveillance is responsible for tracking and reporting SAEs to external and internal monitors. The DCI Safety Team will also manage any external safety reporting required by NIH and Pfizer. The contact for this is Marla Jordan, (919) 681-2351.

11.1 Adverse Events

An adverse event (AE) is any untoward medical occurrence in a subject receiving study therapy and which does not necessarily have a causal relationship with this treatment. For this protocol, the definition of AE also includes worsening of any pre-existing medical condition. An AE can therefore be any unfavorable and unintended or worsening sign (including an abnormal laboratory finding), symptom, or disease temporally associated with the use of study therapy, whether or not related to use of the study therapy. Abnormal laboratory findings without clinical significance (based on the PI's judgment)

should not be recorded as AEs. But laboratory value changes that require therapy or adjustment in prior therapy are considered adverse events.

From the time the subject signs the informed consent form through the End of Study visit (as defined in Section 10.3), all AEs must be recorded in the subject medical record and adverse events case report form. The investigator will follow all reportable events until resolution, a return to baseline condition or stabilization.

AEs will be assessed according to the CTCAE version 4.0. If CTCAE grading does not exist for an AE, the severity of the AE will be graded as mild (1), moderate (2), severe (3), life-threatening (4), or fatal (5).

Attribution of AEs will be indicated as follows:

- Definite: The AE is clearly related to the study therapy
- Probably: The AE is likely related to the study therapy
- Possible: The AE may be related to the study therapy
- Unlikely: The AE is doubtfully related to the study therapy
- Unrelated: The AE is clearly NOT related to the study therapy

11.1.1. AEs of Special Interest

There are not AEs of particular concern or interest in this study because all medications used are FDA approved for smoking cessation.

11.1.2. Reporting of AEs

During each annual IRB review of the protocol, a list of all AEs will be provided to the IRB for review. This report includes whether the AE was likely related to study procedures, whether it impacted subject participation, whether the AE was resolved, and any other action taken.

11.2 Serious Adverse Events

An AE is considered “serious” if in the opinion of the investigator it is one of the following outcomes:

- Fatal
- Life-threatening
- Constitutes a congenital anomaly or birth defect
- A medically significant condition (defined as an event that compromises subject safety or may require medical or surgical intervention to prevent one of the three outcomes above).
- Requires inpatient hospitalization or prolongation of existing hospitalization
- Results in persistent or significant incapacity or substantial disruption to conduct normal life functions.

All SAEs must be followed until resolution, a return to baseline condition, stabilization, or 30 days after the subject’s last dose of study drug whichever occurs first. Any SAEs that are ongoing at the time the clinical database is closed will be reported to the supporting company as unresolved.

11.2.1. Reporting of SAEs

Life-threatening (grade 4 or 5) SAEs, deaths, and unknown reactions or unexpected events that occur in the course of any patient’s treatment of study (from the time of consent) or within 30 days following cessation of treatment are reportable. All SAE’s will be reported to the IRB. The initial report for each SAE or death should include <u>at minimum</u> the following information:

- Protocol # and title
- Patient initials, study identification number, sex, age
- Date the event occurred
- Description of SAE
- Description of patient’s condition
- Indication whether the patient remains of study
- Causality or causal relationship

De-identified source documentation (i.e. discharge notes) must be sent with the SAE Report Form. Follow-up information including severity, action taken, concomitant medications, and outcome should be communicated to Duke as soon as possible using the same forms mentioned above.

As soon as an investigator becomes aware of an AE that meets the definition of serious:

- DCI Safety Surveillance must be notified and they will submit to Pfizer of designee within 24 hours of Investigator's awareness, even if it is not felt to be drug related. Report to Pfizer U.S. Clinical Trial Department (Fax # 866-997-8322)
- The investigator agrees to provide supplementary information requested by Pfizer Drug Safety personnel or designee.

SAEs will be reported by the P.I. in an expedited manner to the Duke University Health System (DUHS) Institutional Review Board (IRB) office, NIDA, and FDA.
SAEs will be reported to the DUHS IRB using the following timelines:

- 24 hours – for an unanticipated study-related death
- 1 week (5 business days) – for an unanticipated problem that is a serious adverse event
- 2 weeks (10 business days) – for an unanticipated problem that does not meet the criteria of a serious adverse event

SAEs will be reported to the NIDA Serious Adverse Event Tracking and Reporting System (SAETRS) within 72 hours. The NIDA Program Official will be kept apprised of IRB actions related to SAEs and any plan to modify the protocol as a result of IRB recommendation will be subject to NIDA approval.

In accordance with applicable regulations, investigators will submit the SAE report to their local IRB according to local IRB institutional guidelines.
Duke as the lead center is responsible for reporting the serious adverse event to the FDA in accordance with 21 CFR 312.32. Any SAE that is possibly related and unexpected must be submitted to the FDA. This will be done by DCI Safety Desk and/or coordinate with study team regulatory coordinator.

- These reports are to be filed utilizing the Form FDA 3500 (MedWatch Form) completed by the DCI Safety Desk.
- The final MedWatch Form must be submitted by the study site to Pfizer within one to two business days of submission to the FDA to allow Pfizer time to cross-report to Pfizer IND.

11.3 Emergency Unblinding of Investigational Treatment

Study staff in direct contact with participants (Clinical Trials Assistants, Study Clinician) will not be blinded to the selection of Varenicline or Nicotine Patch. All study staff in direct contact with participants will be blinded to the randomization to adaptive vs standard treatment algorithms. The Clinical Research Coordinator will have access to participant randomization for emergency un-blinding. In the case that Emergency un-blinding is required, the Principle Investigator will contact the Clinical Research Coordinator directly to obtain the necessary information. Emergency contact information for un-blinding will be the email and cell phone number of Leah Thomas, the study CRC: leah.thomas@duke.edu; 704-798-4498.

11.4 Other Reportable Information

Other reportable information including pregnancy, development of disease or hospitalization for non-study related causes will be reported as AEs or SAEs depending on the severity and assessed as to whether these are related to study procedures.

11.5 Special Warnings and Precautions

There are no special warnings or precautions for this study.

11.6 Stopping Rules

The study will be stopped prior to its completion if: (1) difficulty in study recruitment or retention will significantly impact the ability to evaluate the study endpoints; (2) any new information becomes available during the study that necessitates stopping the study; or (3) other situations occur that might warrant stopping the study. The P.I. will include an assessment of futility in the annual progress report to NIDA and will consult with the study monitors to assess the impact of significant data loss due to problems in recruitment, retention, or data.

11.7 Safety Oversight Committee (SOC)

The SOC is responsible for annual data and safety monitoring of DUHS sponsor-investigator phase I and II, therapeutic interventional studies that do not have an independent Data Safety Monitoring Board (DSMB). The primary focus of the SOC is review of safety data, toxicities and new information that may affect subject safety or efficacy. Annual safety reviews, includes, but may not be limited to review of safety data, enrollment status, stopping rules if applicable, accrual, toxicities, reference literature, and interim analyses as provided by the sponsor-investigator. The SOC oversees the conduct of DUHS sponsor-investigator greater-than-minimal-risk intervention studies that do not have an external monitoring plan, ensuring subject safety and that the protocol is conducted, recorded and reported in accordance with the protocol, standing operating procedures (SOPs), Good Clinical Practice (GCP), and applicable regulatory requirements. The DCI Safety Oversight Committee (SOC) will perform annual reviews on findings from the DCI Monitoring Team visit and additional safety and toxicity data submitted by the Principal Investigator.

11.8 External Data and Safety Monitoring Board (DSMB)

NIDA website describing the criteria for DSMB states "A Data and Safety Monitoring Board (DSMB) is generally required for Phase III clinical trials. For earlier medication trials (Phase I or II and some behavioral clinical trials), a DSMB may be appropriate if the study has multiple clinical sites, is blinded, tests a high-risk intervention, or is conducted in vulnerable populations." According to these criteria, this study should not require a DSMB; it is not a Phase III clinical trial, does not use multiple clinical sites, is not blinded, does not test a high-risk intervention, and is not conducted in a vulnerable population.

12 QUALITY CONTROL AND QUALITY ASSURANCE

12.1 Monitoring

The Duke Cancer Institute (DCI) Monitoring Team will conduct monitoring visits to ensure subject safety and to ensure that the protocol is conducted, recorded, and reported in accordance with the protocol, standard operating procedures, good clinical practice, and applicable regulatory requirements. As specified in the DCI Data and Safety Monitoring Plan, the DCI Monitoring Team will conduct routine monitoring after the third subject is enrolled, followed by annual monitoring of 1 – 3 subjects until the study is closed to enrollment and subjects are no longer receiving study interventions that are more than minimal risk.

Additional monitoring may be prompted by findings from monitoring visits, unexpected frequency of serious and/or unexpected toxicities, or other concerns and may be initiated upon request of DUHS and DCI leadership, the DCI Cancer Protocol Committee, the Safety Oversight Committee (SOC), the sponsor, the Principal Investigator, or the IRB. All study documents must be made available upon request to the DCI Monitoring Team and other authorized regulatory authorities, including but not limited to the National Institute of Health, National Cancer Institute, and the FDA. Every reasonable effort will be made to maintain confidentiality during study monitoring.

12.2 Audits

The Duke School of Medicine Clinical Trials Quality Assurance (CTQA) office may conduct confidential audits to evaluate compliance with the protocol and the principles of GCP. The PI agrees to allow the CTQA auditor(s) direct access to all relevant documents and to allocate his/her time and the time of the study team to the CTQA auditor(s) in order to discuss findings and any relevant issues.

CTQA audits are designed to protect the rights and well-being of human research subjects. CTQA audits may be routine or directed (for cause). Routine audits are selected based upon risk metrics generally geared towards high subject enrollment, studies with limited oversight or monitoring, Investigator initiated Investigational Drugs or Devices, federally-funded studies, high degree of risk (based upon adverse events, type of study, or vulnerable populations), Phase I studies, or studies that involve Medicare populations. Directed audits occur at the directive of the IRB or an authorized Institutional Official.

CTQA audits examine research studies/clinical trials methodology, processes and systems to assess whether the research is conducted according to the protocol approved by the DUHS IRB. The primary purpose of the audit/review is to verify that the standards for safety of human subjects in clinical trials and the quality of data produced by the clinical trial research are met. The audit/review will serve as a quality assurance measure, internal to the institution. Additional goals of such

audits are to detect both random and systemic errors occurring during the conduct of clinical research and to emphasize "best practices" in the research/clinical trials environment.

12.3 Data Management and Processing

12.3.1. Case Report Forms (CRFs)

The eCRF will be the primary data collection document for the study. The CRFs will be updated in a timely manner following acquisition of new source data. Only approved study staff, Principal Investigator, Study Coordinator, and Clinical Trials Assistants, are permitted to make entries or corrections in the CRF.

An audit trail will be maintained automatically by the electronic CRF management system in REDCap and Epic. Designated personnel will complete user training, as required or appropriate per regulations.

12.3.2. Data Management Procedures and Data Verification

Designated personnel using the electronic CRF will have access based on their specific roles in the protocol. Please specify them here.

Completeness of entered data will be checked automatically by the eCRF system, and users will be alerted to the presence of data inconsistencies. Additionally, the Study Coordinator and CTA will cross-reference the data to verify accuracy. Missing or implausible data will be highlighted for the PI requiring appropriate responses (i.e. confirmation of data, correction of data, completion or confirmation that data is not available, etc.).

The database will be reviewed and discussed prior to database closure, and will be closed only after resolution of all remaining queries. An audit trail will be kept of all subsequent changes to the data.

12.3.3. Study Closure

Following completion of the studies, the PI will be responsible for ensuring the following activities:

- Data clarification and/or resolution
- Accounting, reconciliation, and destruction/return of used and unused study drugs, if applicable
- Review of site study records for completeness
- Shipment of all remaining laboratory samples to the designated laboratories.

13 STATISTICAL METHODS AND DATA ANALYSIS

All statistical analysis will be performed under the direction of the statistician designated in key personnel. Any data analysis carried out independently by the investigator must be approved by the statistician before publication or presentation.

13.1 Primary Objective

1. The primary study outcome is comparison of 30-day continuous biochemically confirmed abstinence at 12-weeks post-TQD in participants randomized to adaptive pharmacotherapy algorithm vs. standard-of-care treatment. This will be confirmed by carbon monoxide breath testing with CO < 7 ppm.

13.2 Secondary Objectives

2. To compare 7-day point prevalent biochemically confirmed abstinence rates at 2- and 12-weeks post-TQD in participants randomized to adaptive pharmacotherapy vs. standard-of-care treatment (Varenicline/Nicotine patch).
3. To compare phone-assessed self-reported abstinence at 1, 2, 6, 12, 26 and 52 weeks post-TQD in participants randomized to adaptive vs standard treatment. Phone assessments will be compared at specific time points and also across multiple time points using multi-level modeling.
4. To compare smoking reduction (number of cigarettes per day) at 2 and 12 weeks post TQD in participants randomized to adaptive vs standard treatment.
5. To compare smoking reduction (number of cigarettes per day) on phone-based self-report at 1, 2, 6, 12, 26, and 52 weeks post-TQD in participants randomized to adaptive vs. standard treatment. Phone assessments will be compared at specific time points and also across multiple time points using multi-level modeling.

13.3 Handling of missing values, censoring, and discontinuations

An intent-to-treat approach will be taken in which any subjects lost to follow-up after the point of randomization will be counted as non-abstinent. In addition, a sensitivity analysis will be conducted to determine the impact of "missingness" and smoking at previously observed time-points.

13.4 Statistical Hypothesis, Model, and Method of Analysis

Outcomes will be analyzed using generalized linear regression modeling using an ANOVA-based design; randomization status to both pharmacotherapy and behavioral groups will be denoted using a proxy variable to test primary study effects. An alpha criterion of 0.05 (two-tailed) will be used in all comparisons.

Data Management and Statistical Procedures: All data measures collected during study visits (e.g., smoking history, demographics, standardized tests on nicotine dependence, anxiety, stress etc.) will be captured in the EMR. Phone-based data will be captured using REDCap, an encrypted, HIPAA compliant, Duke Translational Medicine Institute (DTMI)-based data collection system allowing for multi-site data entry, direct participant electronic data upload through interface with computer terminals or tablets, time-based data entry (for use in calendar-based Assessment Visits) and capacity for direct upload into statistical programs SAS, SPSS, R, Excel for analysis. Trained staff (Duke CITI certification) will conduct data integrity processes for REDCap data with download into SAS (SAS Institute, Cary NC).

Multilevel Modeling: Given repeated observations across multiple individuals, many of the analyses in this study will be derived from multilevel modeling (MLM). MLM models hierarchically organized data, in which data at the lowest level of the analysis (e.g., measured at a given time point) can be modeled as a function of time point-specific (Level 1) variables and person-specific variables (Level 2). Unlike repeated-measures ANOVA, MLM is able to also handle imbalanced data with unequal variances across clusters/individuals (Searle S., 1992). In addition to modeling between-person differences in momentary levels of an outcome variable (MLM with intercepts as outcome), MLM is also able to model individual differences in changes in momentary outcomes over time (MLM with intercepts and slopes as outcomes), also known as latent-growth curve modeling. Because MLM procedures are based on maximum likelihood estimation and use all available data, MLM can accommodate data missing at random and is often considered favorable to multiple imputation techniques, which can yield results that vary substantially depending on the imputation model and iteration (D, 2012; L & K, 1998). Nevertheless, missing data will be examined to determine whether missingness is systematic (i.e., associated with individual baseline differences or time-related changes in observed variables). An intent-to-treat approach in which subjects lost to follow-up will be counted as non-abstinent will also be used to analyze univariate outcomes. An alpha criterion of 0.05 (two-tailed) will be used in all comparisons.

Analysis of Primary Outcome: The primary outcome measure is biochemically confirmed 30-day continuous abstinence at the 12-week post-Target Quit Day (TQD) confirmed by carbon monoxide (CO) breath testing with CO < 7ppm. The study hypothesis is that adaptive treatment arms of the study will show higher smoking abstinence at 12-weeks post-quit attempt vs. non-adaptive treatment. An intent-to-treat approach will be taken to analyze primary outcomes such that all subjects who are enrolled in the study will be included in the primary analysis of outcomes. Outcomes will be analyzed using generalized linear regression modeling using an ANOVA-based design. An alpha criterion of 0.05 (two-tailed) will be used in all comparisons. For a conservative estimation of abstinence, subjects lost to follow-up after the point of randomization will be counted as non-abstinent. Following this, however, a sensitivity analysis will be conducted to determine the impact of this assumption, using multiple imputation procedures that model smoking outcomes in terms of "missingness" and smoking at previously observed time-points.

Secondary smoking outcomes: Analysis of secondary smoking outcomes will include comparison of abstinence rates in Adaptive vs. Non-Adaptive groups via self-reported 7 and 30-day continuous abstinence and number of cigarettes smoked per day at 2- and 12-weeks post TQD. Secondary smoking outcomes will be assessed using intent-to-treat principles with use of multiple imputation to account for missing data.

13.2 Exploratory Objectives

Exploration Analysis: The unique aspects of this proposed intervention, related to development of an adaptive approach to smoking cessation treatment is expected to provide a rich database for subsequent exploratory analyses. Accordingly, we will conduct a number of such analyses primarily – but not restricted to – investigating putative associations between primary outcome measures of abstinence, compliance, attrition, with numerous demographic, ecological, clinical, physical, and psychological factors (including but not limited to: age, gender, race, income, education, insurance status, nicotine dependence, physical and psychiatric comorbidity), measured and tested variously across, between, and within conditions. As we are proposing these studies as exploratory, a-priori assumptions on outcomes, although not excluded, are also not mandated. Modeling strategies and details will necessarily be assessed through study outcomes with questions and requisite data not outlined herein.

13.5.1 Key Exploratory Objective Analysis

Patient demographics and other baseline characteristics: The unique aspects of this proposed intervention, related both to development of an adaptive approach to tobacco use treatment and as an exemplar of a large scale translational study into the feasibility and efficacy of service engagement and delivery, is expected to provide a rich database for subsequent exploratory analyses. Accordingly, we will conduct a number of such analyses primarily, but not restricted to, investigating putative associations between primary outcome measures of abstinence, compliance, and attrition with numerous demographic, ecological, clinical, physical, and psychological factors (including but not limited to: age, gender, race, income, education, insurance status, nicotine dependence, physical and psychiatric comorbidity), measured and tested variously across, between, and within conditions. As we are proposing these studies as exploratory, a-priori assumptions on outcomes, although not excluded, are also not mandated. Modeling strategies and details will necessarily be dictated by the structures of both the questions and the requisite data and, hence, are not outlined herein.

Assessment of potential moderators of primary effect: The following constructs will be assessed as potential predictors of abstinence: demographics (age, gender, race, education), smoking history and heaviness, financial status, dependence (measured by FTND), urge rating (measured by MPSS), depression (measured by PHQ-2), anxiety (measured by GAD-2), alcohol use (measured by AUDIT-C), stress (measured by PSS-4), and self-efficacy questions. Constructs that demonstrate an association with abstinence will be analyzed for potential moderation. Tests for differential associations of effects of baseline characteristics by randomization status on week-12 smoking abstinence will be based on interaction terms crossing randomization status (group) with the tested (putative) baseline predictor. Significance of the estimated coefficient associated with the interaction term would indicate that the effect of the tested predictor on abstinence varied differentially by treatment assignment. For each baseline predictor, we hypothesize that baseline will be equivalent across all randomized arms. In a second series of tests, the above protocol will be used to test for differential rates of change in abstinence associated with putative constructs (scales): urge (MPSS-2), stress (PSS-4), depression (PHQ-2), anxiety (GAD-2). As above, the primary test statistic will be based on interaction term crossing the tested scale with group.

Assessment of potential mediators of primary effect: The following constructs will be assessed to determine whether assignment to pharmacotherapy group are associated with change in these constructs pre-post treatment: urge rating (MPSS), depression (PHQ-2), anxiety (GAD-2), stress (PSS-4), self-efficacy. Positive findings for candidate measures would also recommend them for subsequent testing as mediators using formal mediation paradigms (VanderWeele TJ, 2014). These methods now supersede the methodology advocated by Baron and Kenny (1986), and are based on a four-way decomposition of the effects of exposure on an outcome in the presence of a putative mediator. That is, using standard statistical procedures (i.e. regression), effects are decomposed into four components: i) an effect of the exposure in the absence of the mediator, ii) an additive effect where both exposure and the mediator affect the outcome but the mediator is not affected by exposure, iii) the mediated interaction where exposure has an effect on the mediator, and iv) a pure mediated effect where exposure causes the mediator to have an effect although subsequent presence of the mediator is not required. These components are subsequently used to decompose total estimated effect into the proportions of that

effect due to neither mediation nor interaction, exclusively to interaction, to a combined effect including both mediation and interaction, and exclusively to interaction.

Economic Analysis: An economic analysis of Adaptive vs. Non-Adaptive pharmacotherapy will be conducted. Dr. Santanu Datta, PhD, MBA, a Duke University Health Economist with expertise in cost-benefit analysis for healthcare system program operation will conduct this analysis and oversee prospective data-collection on cost and time for all necessary TSCP operations to generate a prospective economic analysis of adaptive vs. non-adaptive treatment to identify cost-benefit outcomes from the perspective of smokers, healthcare systems, insurance companies, and employers. Cost assessments of adaptive treatment will include: 1) Descriptive statistics to compare overall cost of medications for adaptive vs. non-adaptive treatment. 2) ANOVA modeling to analyze cost of medications per abstinent smoker comparing adaptive vs. non-adaptive treatment. 3) ANOVA modeling to analyze the cost of prescribed medications as related to medication non-adherence and attrition. 4) Mediation analysis on how randomization status affects medication adherence and how that relationship is mediated by income.

13.3 Interim Analyses

1. There will be an interim analysis after completion of the 12 week visit for all 300 participants. This will include data from all Clinical Assessment Visits, but exclude data from phone visits out to 1 year and will be sufficient for early initiation of manuscript preparation.

13.4 Sample Size Calculation

Outcomes: The primary outcome for this study is biochemically confirmed 30-day continuous smoking abstinence at 12 weeks post TQD across all enrolled participants (intent-to-treat). Secondary outcomes include smoking abstinence at other time points (2 week visit), smoking reduction from baseline to follow-up visits, assessment of baseline variables as potential moderators of smoking behavior, assessment of psychological variables as potential mediators of smoking behavior, and assessment of economic outcomes. The sample size for this study was calculated based on effect sizes from prior studies on Varenicline and nicotine patch. Abstinence within these two arms will be assessed as secondary outcomes and are not expected to reach significance on all secondary outcomes. The study is powered to reach significance on the primary outcome (abstinence across the two arms combined).

Nicotine Patch Based Treatment (secondary outcome): Power calculation for Nicotine Patch Adaptive Treatment vs. Nicotine Patch Standard Treatment is based on abstinence rates observed in prior P50-funded trials. In one trial (Rose et al 2013), when participants received Nicotine Patch prior to their quit attempt, 41.2% showed >50% reduction in cigarettes-per-day (considered Patch Responders) whereas 58.8% did not show >50% reduction in cigarettes-per-day (were considered Patch Non-Responders). "Patch Responders" demonstrated biochemically-confirmed 4-week continuous abstinence at the 11-week post-quit attempt of 59.2%, whereas "Patch Non-Responders," when left on Nicotine Patch alone showed an abstinence rate of 16.0%. When however, the Patch Non-Responders were given Bupropion in addition to Nicotine Patch, they showed an increased abstinence of 28.3%. If our study were to show similar effect sizes as this prior study, the adaptive treatment group with 41.2% responders (abstinence rate = 59.2%), and 58.8% non-responders given Nicotine Patch + Bupropion (abstinence) rate = 28.3%), should show an abstinence rate for responders + non-responders of 41.0%. Abstinence rates for Nicotine Patch Standard Treatment in several CSC trials using the similar recruitment and exclusion criteria range from 17-26%. With a conservative assumption that we will see the higher abstinence rates in our controls (e.g. 26%), our two Nicotine Patch-Based Treatment groups should show abstinence rates of **41.2% vs. 26%.** Sample size calculation using 2-tail test with continuity correction assuming p = 0.05 and power = 80%, yields a minimum sample size of **298** in the nicotine patch arm. This study is designed with 150 in the Nicotine Patch arm and so it underpowered to reach this secondary outcome.

Varenicline-Based Treatment (secondary outcome): Power calculation for Varenicline Based Adaptive treatment vs. Varenicline Standard Treatment is based on abstinence rates observed in prior P50-funded trials as well as other published studies. To estimate the effect of adaptive treatment, a critical consideration is that 4 week Varenicline pre-cessation treatment without adaptation was superior to starting 1 week prior to quit day (Hajek et al 2011). Abstinence rate were 20.8% 1-week pre-quit start time. Hajek further showed that for participants starting 4 weeks prior to the Quit day, 35.4% of participants showed >50% reduction in cigs/day with an abstinence rate of 66.7% whereas 64.6% of participants failed to show >50% reduction in cigs/day and had an abstinence rate of 22.6%. The weighted average of these groups = 38.2%. Sample size calculation using **38.2% vs. 20.8%** with 2-tail test with continuity correction assuming p = 0.05 and power = 80%, yields a minimum sample size of **208** within the Varenicline arm alone. This study is designed with 150 in the Varenicline arm and so it underpowered to reach this secondary outcome.

Full Sample (Primary Outcome): Based on these calculations, a sample size of 300 for each of these comparisons should be sufficient to demonstrate significant differences between randomized arms (150 vs. 150). The Primary Outcome is a comparison of 12-week post-TQD smoking abstinence across the study sample (N=300). As stated above, the nicotine patch arm will require n=298 to reach significance, and the varenicline arm will require n=208 to reach significance. Neither arm (150 each) is powered to reach significance. The two arms together (n=300) are powered to reach primary outcomes. For the control group (nicotine patch arm = 26.0%; varenicline arm = 20.8%), an equally weighted sample would result in an abstinence rate of **23.4%**. For the adaptive treatment group (nicotine patch arm = 41.2%; varenicline arm = 38.2%), an equally weighted sample would result in abstinence rate of **39.7%**. A sample size calculation using 2-tail test with continuity correction assuming p = 0.05 and power = 80% results in sample size of **248.** With 300 participants this study as power of 87% power for showing significant results on our primary outcome.

14 ADMINISTRATIVE AND ETHICAL CONSIDERATIONS

14.1 Regulatory and Ethical Compliance

This protocol was designed and will be conducted and reported in accordance with the International Conference on Harmonization (ICH) Harmonized Tripartite Guidelines for Good Clinical Practice, the Declaration of Helsinki, and applicable federal, state, and local regulations.

14.2 DUHS Institutional Review Board and DCI Cancer Protocol Committee

The protocol, informed consent form, advertising material, and additional protocol-related documents must be submitted to the DUHS Institutional Review Board (IRB) and DCI Cancer Protocol Committee (CPC) for review. The study may be initiated only after the Principal Investigator has received written and dated approval from the CPC and IRB.

The Principal Investigator must submit and obtain approval from the IRB for all subsequent protocol amendments and changes to the informed consent form. The CPC should be informed about any protocol amendments that potentially affect research design or data analysis (i.e. amendments affecting subject population, inclusion/exclusion criteria, agent administration, statistical analysis, etc.).

The Principal Investigator must obtain protocol re-approval from the IRB within 1 year of the most recent IRB approval. The Principal Investigator must also obtain protocol re-approval from the CPC within 1 year of the most recent IRB approval, for as long as the protocol remains open to subject enrollment.

14.3 Informed Consent

The informed consent form must be written in a manner that is understandable to the subject population. Prior to its use, the informed consent form must be approved by the IRB.

The Principal Investigator or authorized key personnel will discuss with the potential subject the purpose of the research, methods, potential risks and benefits, subject concerns, and other study-related matters. This discussion will occur in a location that ensures subject privacy and in a manner that minimizes the possibility of coercion. Appropriate accommodations will be made available for potential subjects who cannot read or understand English or are visually impaired. Potential subjects will have the opportunity to contact the Principal investigator or authorized key personnel with questions, and will be given as much time as needed to make an informed decision about participation in the study.

Before conducting any study-specific procedures, the Principal Investigator must obtain written informed consent from the subject or a legally acceptable representative. The original informed consent form will be stored with the subject's study records, and a copy of the informed consent form will be provided to the subject. The Principal Investigator is responsible for asking the subject whether the subject wishes to notify his/her primary care physician about participation in the study. If the subject agrees to such notification, the Principal Investigator will inform the subject's primary care physician about the subject's participation in the clinical study.

14.4 Study Documentation

Study documentation includes but is not limited to source documents, case report forms (CRFs), monitoring logs, appointment schedules, study team correspondence with sponsors or regulatory bodies/committees, and regulatory documents that can be found in the DCI-mandated "Regulatory Binder", which includes but is not limited to signed

protocol and amendments, approved and signed informed consent forms, FDA Form 1572, CAP and CLIA laboratory certifications, and clinical supplies receipts and distribution records.

Source documents are original records that contain source data, which is all information in original records of clinical findings, observations, or other activities in a clinical trial necessary for the reconstruction and evaluation of the trial. Source documents include but are not limited to hospital records, clinical and office charts, laboratory notes, memoranda, evaluation checklists, pharmacy dispensing records, recorded data from automated instruments, copies or transcriptions certified after verification as being accurate copies, microfiches, photographic negatives, microfilm or magnetic media, x-rays, subject files, and records kept at the pharmacy, at the laboratories and at medico-technical departments involved in the clinical trial. When possible, the original record should be retained as the source document. However, a photocopy is acceptable provided that it is a clear, legible, and an exact duplication of the original document. An electronic case report form (CRF) will be the primary data collection document for the study. The CRFs will be updated within two weeks of acquisition of new source data. Only approved study staff (please specify names and/or titles of authorized individuals as stated in key personnel), are permitted to make entries, changes, or corrections in the CRF. For paper CRFs, errors will crossed out with a single line, and this line will not obscure the original entry. Changes or corrections will be dated, initialed, and explained (if necessary). The Principal Investigator or authorized key personnel will maintain a record of the changes and corrections. For electronic CRFs, an audit trail will be maintained by the electronic CRF management systems, REDCap and Epic Dashboard.

14.5 Privacy, Confidentiality, and Data Storage

The Principal Investigator will ensure that subject privacy and confidentiality of the subject's data will be maintained. Research Data Security Plans (RDSPs) will be approved by the appropriate institutional Site Based Research group. To protect privacy, every reasonable effort will be made to prevent undue access to subjects during the course of the study. Prospective participants will be consented in an exam room where it is just the research staff, the patient and his family, if desired. For all future visits, interactions with research staff (study doctor and study coordinators) regarding research activities will take place in a private exam room. All research related interactions with the participant will be conducted by qualified research staff who are directly involved in the conduct of the research study.

To protect confidentiality, subject files in paper format will be stored in secure cabinets under lock and key accessible only by the research staff. Subjects will be identified only by a unique study number and subject initials. Electronic records of subject data will be maintained using dedicated databases: eResearch, REDCap, and Epic Dashboard, which is housed in an encrypted and password-protected (required: please specify storage device, i.e. laptop computer, desktop computer, DCI file server). Access to electronic databases will be limited to study staff. Subject data may be stored temporarily on encrypted and password-protected portable memory devices such as flash drives and external hard drives, but only when absolutely necessary. Data stored on portable memory devices will be de-identified. Subject data will be deleted from the portable memory device at the earliest opportunity. The security and viability of the IT infrastructure will be managed by the DCI and/or Duke Medicine. Upon completion of the study, research records will be archived and handled per DUHS HRPP policy. Subject names or identifiers will not be used in reports, presentations at scientific meetings, or publications in scientific journals.

14.6 Data and Safety Monitoring

Data and Safety Monitoring will be performed in accordance with the DCI Data and Safety Monitoring Plan. For a more detailed description of the DSMP for this protocol, refer to Section 11 (Sections 11.7 and 11.8 in particular) along with section 12.

14.7 Protocol Amendments

All protocol amendments must be initiated by the Principal Investigator and approved by the IRB prior to implementation. IRB approval is not required for protocol changes that occur to protect the safety of a subject from an immediate hazard. However, the Principal Investigator must inform the IRB and all other applicable regulatory agencies of such action immediately.

14.8 Records Retention

The Principal Investigator will maintain study-related records for at least six years after study completion.

15 REFERENCES

Alterman, A. I., Gariti, P., & Mulvaney, F. (2001). Short- and long-term smoking cessation for three levels of intensity of behavioral treatment. *Psychol Addict Behav, 15*(3), 261-264.

Barnett, P. G., Wong, W., Jeffers, A., Munoz, R., Humfleet, G., & Hall, S. (2014). Cost-effectiveness of extended cessation treatment for older smokers. *Addiction, 109*(2), 314-322. doi:10.1111/add.12404

Berman, M., Crane, R., Seiber, E., & Munur, M. (2014). Estimating the cost of a smoking employee. *Tob Control, 23*(5), 428-433. doi:10.1136/tobaccocontrol-2012-050888

Brandon, T. H. (2001). Behavioral tobacco cessation treatments: yesterday's news or tomorrow's headlines? *J Clin Oncol, 19*(18 Suppl), 64S-68S.

Bunn, W. B., 3rd, Stave, G. M., Downs, K. E., Alvir, J. M., & Dirani, R. (2006). Effect of smoking status on productivity loss. *J Occup Environ Med, 48*(10), 1099-1108. doi:10.1097/01.jom.0000243406.08419.74

Burton, W. N., Chen, C. Y., Conti, D. J., Schultz, A. B., Pransky, G., & Edington, D. W. (2005). The association of health risks with on-the-job productivity. *J Occup Environ Med, 47*(8), 769-777.

Bush, K., Kivlahan, D. R., McDonell, M. B., Fihn, S. D., & Bradley, K. A. (1998). The AUDIT alcohol consumption questions (AUDIT-C): an effective brief screening test for problem drinking. Ambulatory Care Quality Improvement Project (ACQUIP). Alcohol Use Disorders Identification Test. *Archives of Internal Medicine, 158*(16), 1789-1795.

Cahill, K., & Perera, R. (2008). Competitions and incentives for smoking cessation. *Cochrane Database Syst Rev*(3), CD004307. doi:10.1002/14651858.CD004307.pub3

Cahill, K., Stevens, S., Perera, R., & Lancaster, T. (2013). Pharmacological interventions for smoking cessation: an overview and network meta-analysis. *Cochrane Database Syst Rev, 5*, CD009329. doi:10.1002/14651858.CD009329.pub2

CDC. (2008). Centers for Disease Control and Prevention : Smoking-attributable mortality, years of potential life lost, and productivity losses—United States, 2000-2004. *MMWR. Morbidity and mortality weekly report, 57*(45), 1226.

Christenhusz, L., Pieterse, M., Seydel, E., & van der Palen, J. (2007). Prospective determinants of smoking cessation in COPD patients within a high intensity or a brief counseling intervention. *Patient Education and Counseling, 66*(2), 162-166. doi:10.1016/j.pec.2006.11.006

Cohen, S., Kamarck, T., & Mermelstein, R. (1983). A global measure of perceived stress. *J Health Soc Behav, 24*(4), 385-396.

Cokkinides, V., Bandi, P., McMahon, C., Jemal, A., Glynn, T., & Ward, E. (2009). Tobacco control in the United States--recent progress and opportunities. *CA Cancer J Clin, 59*(6), 352-365. doi:10.3322/caac.20037

Cooper, S., Lewis, S., Thornton, J. G., Marlow, N., Watts, K., Britton, J., . . . Pregnancy Trial, T. (2014). The SNAP trial: a randomised placebo-controlled trial of nicotine replacement therapy in pregnancy--clinical effectiveness and safety until 2 years after delivery, with economic evaluation. *Health Technol Assess, 18*(54), 1-128. doi:10.3310/hta18540

D, A. P. (2012). *Handling missing data by maximum likelihood*. Paper presented at the SAS Global Forum, Florida. http://statisticalhorizons.com/wp-content/uploads/MissingDataByML.pdf

Dornelas, E. A., Magnavita, J., Beazoglou, T., Fischer, E. H., Oncken, C., Lando, H., . . . Gregonis, E. (2006). Efficacy and cost-effectiveness of a clinic-based counseling intervention tested in an ethnically diverse sample of pregnant smokers. *Patient Education and Counseling, 64*(1-3), 342-349. doi:10.1016/j.pec.2006.03.015

Drovandi, A. D., Chen, C. C., & Glass, B. D. (2016). Adverse Effects Cause Varenicline Discontinuation: A Meta-Analysis. *Curr Drug Saf, 11*(1), 78-85.

Ebbert, J. O. (2013). Varenicline and combination nicotine replacement therapy are the most effective pharmacotherapies for treating tobacco use. *Evid Based Med, 18*(6), 212-213. doi:10.1136/eb-2013-101462

Eddy, D. (2009). David Eddy ranks the tests. *Harv Health Lett.1992;(special suppl):10-1. medicine, 360*(7), 699-709. doi:10.1056/NEJMsa0806819

Evins, A. E., Mays, V. K., Rigotti, N. A., Tisdale, T., Cather, C., & Goff, D. C. (2001). A pilot trial of bupropion added to cognitive behavioral therapy for smoking cessation in schizophrenia. *Nicotine Tob Res, 3*(4), 397-403. doi:10.1080/14622200110073920

Fagerstrom, K., & Hughes, J. (2008). Varenicline in the treatment of tobacco dependence. *Neuropsychiatr Dis Treat, 4*(2), 353-363.

Fiore, M. C. (2008). Treating tobacco use and dependence: an introduction to the US Public Health Service Clinical Practice Guideline. *Respir Care, 45*(10), 1196-1199.

Giles, E. L., Robalino, S., McColl, E., Sniehotta, F. F., & Adams, J. (2014). The effectiveness of financial incentives for health behaviour change: systematic review and meta-analysis. *PLoS ONE, 9*(3), e90347. doi:10.1371/journal.pone.0090347

GlaxoSmithKline. (2014). ZYBAN (bupropion hydrochloride) Sustained-Release Tablets. Retrieved from https://www.gsksource.com/pharma/content/dam/GlaxoSmithKline/US/en/Prescribing_Information/Zyban/pdf/ZYBAN-PI-MG.PDF

Gordon, L., Graves, N., Hawkes, A., & Eakin, E. (2007). A review of the cost-effectiveness of face-to-face behavioural interventions for smoking, physical activity, diet and alcohol. *Chronic Illn, 3*(2), 101-129. doi:10.1177/1742395307081732

Halpern, M. T., Shikiar, R., Rentz, A. M., & Khan, Z. M. (2001). Impact of smoking status on workplace absenteeism and productivity. *Tob Control, 10*(3), 233-238.

Heatherton, T. F., Kozlowski, L. T., Frecker, R. C., & Fagerstrom, K. O. (1991). The Fagerstrom Test for Nicotine Dependence: a revision of the Fagerstrom Tolerance Questionnaire. *Br J Addict, 86*(9), 1119-1127.

Hughes, J., Lindgren, P., Connett, J., Nides, M., & Lung Health, S. (2004). Smoking reduction in the Lung Health Study. *Nicotine Tob Res, 6*(2), 275-280. doi:10.1080/14622200410001676297

Hughes, J. R., Stead, L. F., Hartmann-Boyce, J., Cahill, K., & Lancaster, T. (2014). Antidepressants for smoking cessation. *Cochrane Database Syst Rev, 1*, CD000031. doi:10.1002/14651858.CD000031.pub4

Irvin, J. E., Hendricks, P. S., & Brandon, T. H. (2003). The increasing recalcitrance of smokers in clinical trials II: Pharmacotherapy trials. *Nicotine Tob Res, 5*(1), 27-35.

Kroenke, K., Spitzer, R. L., & Williams, J. B. (2003). The Patient Health Questionnaire-2: validity of a two-item depression screener. *Med Care, 41*(11), 1284-1292. doi:10.1097/01.MLR.0000093487.78664.3C

Kroenke, K., Spitzer, R. L., & Williams, J. B. W. (2001). The PHQ-9: Validity of a Brief Depression Severity Measure. *Journal of General Internal Medicine, 16*(9), 606-613. doi:10.1046/j.1525-1497.2001.016009606.x

L, S. J., & K, O. M. (1998). Multiple imputation for multivariate missing-data problems: a data analyst's perspective. *Multivariate Behavioral Research, 33*, 545-571.

Mihalak, K. B., Carroll, F. I., & Luetje, C. W. (2006). Varenicline is a partial agonist at alpha4beta2 and a full agonist at alpha7 neuronal nicotinic receptors. *Mol Pharmacol, 70*(3), 801-805. doi:10.1124/mol.106.025130

Mills, E. J., Wu, P., Lockhart, I., Thorlund, K., Puhan, M., & Ebbert, J. O. (2012). Comparisons of high-dose and combination nicotine replacement therapy, varenicline, and bupropion for smoking cessation: a systematic review and multiple treatment meta-analysis. *Ann Med, 44*(6), 588-597. doi:10.3109/07853890.2012.705016

Molero, Y., Lichtenstein, P., Zetterqvist, J., Gumpert, C. H., & Fazel, S. (2015). Varenicline and risk of psychiatric conditions, suicidal behaviour, criminal offending, and transport accidents and offences: population based cohort study. *BMJ, 350*, h2388. doi:10.1136/bmj.h2388

Nides, M., Leischow, S., Sarna, L., & Evans, S. E. (2007). Maximizing smoking cessation in clinical practice: pharmacologic and behavioral interventions. *Prev Cardiol, 10*(2 Suppl 1), 23-30.

Orleans, C. T. (2007). Increasing the demand for and use of effective smoking-cessation treatments reaping the full health benefits of tobacco-control science and policy gains--in our lifetime. *Am J Prev Med, 33*(6 Suppl), S340-348. doi:10.1016/j.amepre.2007.09.003

Pancani, L., D'Addario, M., Cappelletti, E. R., Greco, A., Monzani, D., & Steca, P. (2015). Smoking behavior: a cross-sectional study to assess the dimensionality of the brief Wisconsin inventory of smoking dependence motives and identify different typologies among young daily smokers. *Nicotine Tob Res, 17*(1), 98-105. doi:10.1093/ntr/ntu143

Papadakis, S., Gharib, M., Hambleton, J., Reid, R. D., Assi, R., & Pipe, A. L. (2014). Delivering evidence-based smoking cessation treatment in primary care practice: experience of Ontario family health teams. *Can Fam Physician, 60*(7), e362-371.

Pfizer. (2014). CHANTIX- varenicline tartrate tablet, film coated CHANTIX- varenicline tartrate 	 . Retrieved from http://labeling.pfizer.com/showlabeling.aspx?id=557#section-2

Piper, M. E., Smith, S. S., Schlam, T. R., Fiore, M. C., Jorenby, D. E., Fraser, D., & Baker, T. B. (2009). A randomized placebo-controlled clinical trial of 5 smoking cessation pharmacotherapies. *Arch Gen Psychiatry, 66*(11), 1253-1262. doi:10.1001/archgenpsychiatry.2009.142

Plummer, F., Manea, L., Trepel, D., & McMillan, D. (2016). Screening for anxiety disorders with the GAD-7 and GAD-2: a systematic review and diagnostic metaanalysis. *General Hospital Psychiatry, 39*(Supplement C), 24-31. doi:https://doi.org/10.1016/j.genhosppsych.2015.11.005

Richmond, R., & Zwar, N. (2003). Review of bupropion for smoking cessation. *Drug Alcohol Rev, 22*(2), 203-220. doi:10.1080/09595230100100642

Ronckers, E. T., Groot, W., & Ament, A. J. (2005). Systematic review of economic evaluations of smoking cessation: standardizing the cost-effectiveness. *Med Decis Making, 25*(4), 437-448. doi:10.1177/0272989X05278431

Rose, J. E. (2011). Nicotine preloading: the importance of a pre-cessation reduction in smoking behavior. *Psychopharmacology (Berl), 217*(3), 453-454. doi:10.1007/s00213-011-2350-0

Rose, J. E., & Behm, F. M. (2013). Adapting smoking cessation treatment according to initial response to precessation nicotine patch. *Am J Psychiatry, 170*(8), 860-867. doi:10.1176/appi.ajp.2013.12070919

Rose, J. E., & Behm, F. M. (2014). Combination treatment with varenicline and bupropion in an adaptive smoking cessation paradigm. *Am J Psychiatry, 171*(11), 1199-1205. doi:10.1176/appi.ajp.2014.13050595

Rose, J. E., Herskovic, J. E., Behm, F. M., & Westman, E. C. (2009). Precessation treatment with nicotine patch significantly increases abstinence rates relative to conventional treatment. *Nicotine Tob Res, 11*(9), 1067-1075. doi:10.1093/ntr/ntp103

Ruger, J. P., & Lazar, C. M. (2012). Economic evaluation of pharmaco- and behavioral therapies for smoking cessation: a critical and systematic review of empirical research. *Annu Rev Public Health, 33*, 279-305. doi:10.1146/annurev-publhealth-031811-124553

Ruger, J. P., Weinstein, M. C., Hammond, S. K., Kearney, M. H., & Emmons, K. M. (2008). Cost-effectiveness of motivational interviewing for smoking cessation and relapse prevention among low-income pregnant women: a randomized controlled trial. *Value Health, 11*(2), 191-198. doi:10.1111/j.1524-4733.2007.00240.x

Searle S., C. G., McCulloch C. (1992). *Variance Components*. New York: John Wiley & Sons, Inc.

Sharples, C. G., & Wonnacott, S. (2001). Neuronal nicotinic receptors. *Tocris Reviews, 19*(October 2001), 1-12.

Shiffman, S., Sweeney, C. T., & Dresler, C. M. (2005). Nicotine patch and lozenge are effective for women. *Nicotine Tob Res, 7*(1), 119-127. doi:10.1080/14622200412331328439

Siu, E. C., & Tyndale, R. F. (2007). Non-nicotinic therapies for smoking cessation. *Annu Rev Pharmacol Toxicol, 47*, 541-564. doi:10.1146/annurev.pharmtox.47.120505.105354

Skinner, H. A. (1982). The drug abuse screening test. *Addictive Behaviors, 7*(4), 363-371.

Slemmer, J. E., Martin, B. R., & Damaj, M. I. (2000). Bupropion is a nicotinic antagonist. *J Pharmacol Exp Ther, 295*(1), 321-327.

Stapleton, J., West, R., Hajek, P., Wheeler, J., Vangeli, E., Abdi, Z., . . . Sutherland, G. (2013). Randomized trial of nicotine replacement therapy (NRT), bupropion and NRT plus bupropion for smoking cessation: effectiveness in clinical practice. *Addiction, 108*(12), 2193-2201. doi:10.1111/add.12304
Stead, L. F., & Lancaster, T. (2012). Combined pharmacotherapy and behavioural interventions for smoking cessation. *Cochrane Database Syst Rev, 10*, CD008286. doi:10.1002/14651858.CD008286.pub2
Sussman, S., Valente, T. W., Rohrbach, L. A., Skara, S., & Pentz, M. A. (2006). Translation in the health professions: converting science into action. *Eval Health Prof, 29*(1), 7-32. doi:10.1177/0163278705284441
Taly, A., Corringer, P. J., Guedin, D., Lestage, P., & Changeux, J. P. (2009). Nicotinic receptors: allosteric transitions and therapeutic targets in the nervous system. *Nat Rev Drug Discov, 8*(9), 733-750. doi:10.1038/nrd2927
USDHHS. (2014). *United States. Public Health Service. How tobacco smoke causes disease : the biology and behavioral basis for smoking-attributable disease : a report of the Surgeon General*. Rockville, MD
Washington, DC: U.S. Dept. of Health and Human Services, Public Health Service
For sale by the Supt. of Docs., U.S. G.P.O.
USSGsACo. (1964). *United States. Surgeon General's Advisory Committee on Smoking and Health: Smoking and health : report of the advisory committee to the Surgeon General of the Public Health Service*. Washington, D.C.: U.S. Dept. of Health, Education, and Welfare For sale by the Supt. of Docs., U.S. G.P.O.
Wagena, E. J., Knipschild, P. G., Huibers, M. J., Wouters, E. F., & van Schayck, C. P. (2005). Efficacy of bupropion and nortriptyline for smoking cessation among people at risk for or with chronic obstructive pulmonary disease. *Archives of Internal Medicine, 165*(19), 2286-2292. doi:10.1001/archinte.165.19.2286
Wagena, E. J., van der Meer, R. M., Ostelo, R. J., Jacobs, J. E., & van Schayck, C. P. (2004). The efficacy of smoking cessation strategies in people with chronic obstructive pulmonary disease: results from a systematic review. *Respir Med, 98*(9), 805-815.
Warner, K. E., & Burns, D. M. (2003). Hardening and the hard-core smoker: concepts, evidence, and implications. *Nicotine Tob Res, 5*(1), 37-48.
Warner, K. E., Mendez, D., & Smith, D. G. (2004). The financial implications of coverage of smoking cessation treatment by managed care organizations. *Inquiry, 41*(1), 57-69.
West, R., & Hajek, P. (2004). Evaluation of the mood and physical symptoms scale (MPSS) to assess cigarette withdrawal. *Psychopharmacology (Berl), 177*(1-2), 195-199. doi:10.1007/s00213-004-1923-6
Whitlock, E. P., Orleans, C. T., Pender, N., & Allan, J. (2002). Evaluating primary care behavioral counseling interventions: an evidence-based approach. *Am J Prev Med, 22*(4), 267-284.
WHO. (2002). World Health Organization. The Tobacco Atlas 2002. Retrieved from http://www.who.int/tobacco/media/en/title.pdf

16 APPENDICES

Appendix 1: Smoking Related Medical History

MEDICAL HISTORY pg. l

Major Medical Conditions

Have you had any of the following conditions?

- O Yes O No Uncontrolled high blood pressure (Hypertension) or hypotension with symptoms (<90/60)
- O Yes O No Heart attack OR heart disease diagnosis by coronary angiogram
- O Yes O No Problems with heart valves such as regurgitation, stenosis, or artificial valve
- O Yes O No Heart rhythm problem such as atrial fibrillation, tachycardia, or pacemaker
- O Yes O No Chest pains related to the heart in the last month
- O Yes O No Heart failure
- O Yes O No Skin problems requiring medication
- O Yes O No Liver cirrhosis (with jaundice or swollen abdomen)
- O Yes O No Liver problems other than cirrhosis (e.g. hepatitis, fatty liver)
- O Yes O No Kidney failure requiring dialysis
- O Yes O No Chronic Kidney Disease not requiring dialysis
- O Yes O No Chronic diarrhea due to Irritable Bowel Syndrome, Crohn's Disease, or Inflammatory Bowel
- O Yes O No Stomach/Duodenal Ulcer (Gastrointestinal Ulcer)
- O Yes O No Chronic Bronchitis (cough every morning)
- O Yes O No Chronic Obstructive Pulmonary Disease (COPD) (Emphysema)
- O Yes O No Use of oxygen
- O Yes O No Asthma
- O Yes O No Other chronic lung disorder such as Tuberculosis, Pulmonary Fibrosis, Sarcoid, or other
- O Yes O No Stroke/TIA (mini-stroke)/brain tumor
- O Yes O No Seizure disorder
- O Yes O No Regular headaches or migraines that occur more than one per week
- O Yes O No Unexplained fainting spells
- O Yes O No Insomnia requiring medications
- O Yes O No Other neurologic conditions
- O Yes O No Problems giving blood samples
- O Yes O No Blood disorder or Anemia requiring iron
- O Yes O No Rheumatic Disease such as Rheumatoid Arthritis, Fibromyalgia, or other
- O Yes O No Sinusitis/Seasonal allergies
- O Yes O No Diabetes with uncontrolled blood sugar (>200 more than once/week) or on insulin
- O Yes O No Thyroid disease/condition
- O Yes O No Current cancer or treatment for cancer in the past 6 months
- O Yes O No Depression/Anxiety/Bipolar disorder/psychiatric hospitalization in the past 3 months
- O Yes O No Suicidal ideation (thinking about ways to commit suicide) within the past 10 years
- O Yes O No Suicide attempt during the last year
- O Yes O No Schizophrenia
- O Yes O No Post-Traumatic Stress Disorder (PTSD)
- O Yes O No Other Psychiatric problems (Borderline, Schizoaffective, Hypomania, ADHD)
- O Yes O No Chronic infections syndrome such as HIV, CMV, Epstein Barr
- O Yes O No Anorexia or bulimia

○ Yes ○ No	Undergoing acute discontinuation of alcohol, benzodiazepines, barbiturates, or antiepileptic drugs

Please list any illnesses that caused you to miss work or have interrupted your life this past year:

1. ______________________________ Year: __________
2. ______________________________ Year: __________
3. ______________________________ Year: __________
4. ______________________________ Year: __________
5. ______________________________ Year: __________

Please list any hospitalizations in the past 10 years. If possible, include the year:

1. ______________________________ Year: __________
2. ______________________________ Year: __________
3. ______________________________ Year: __________
4. ______________________________ Year: __________
5. ______________________________ Year: __________

Please list any serious injuries or accidents. If possible, include the year:

1. ______________________________ Year: __________
2. ______________________________ Year: __________
3. ______________________________ Year: __________
4. ______________________________ Year: __________
5. ______________________________ Year: __________

Please list any surgeries or major procedures. If possible, include the year:

1. ______________________________ Year: __________
2. ______________________________ Year: __________
3. ______________________________ Year: __________
4. ______________________________ Year: __________
5. ______________________________ Year: __________

Women Only:

Date of last menstrual cycle: ___________________
Are you menstruating regularly? ○ Yes ○ No
Are you post-menopausal (natural or from surgery)? ○ Yes ○ No

Family History

Has any first degree family member (child, parent, or sibling) had any of the following illnesses?

Illness	Which family member?
Anemia or Blood Disease	

Cancer	
Diabetes	
Heart Disease	
High Blood Pressure	
Severe Mental Illness	
Stroke	
Substance abuse (alcohol, tobacco, or other)	
Other serious illness	

Medications

Please list any medications you are allergic to:

1.	2.	3.

Please list all medications you are now taking or have used in the last month (include over-the-counter drugs, vitamins, and especially prescriptions). It is okay to skip dates for long-term medications:

Name of Medication	Dosage	Start Date	Stop Date	Prescribed for what problem?
1.				
2.				
3.				
4.				
5.				
6.				
7.				
8.				

Are you taking Linezolid or intravenous methylene blue? ○ **Yes** ○ **No**
Have you used monoamine oxidase inhibitors (MAOi) in the past 14 days? ○ **Yes** ○ **No**
Have you used experimental or investigational drugs in the past 30 days? ○ **Yes** ○ **No**

Smoking Cessation Medications

For each of the following, mark if you have used the medication, experienced any side effects, allergy or intolerance with usage, or had to stop taking the medication due to side effects:

	Not used	Used	Side Effects	Stopped due to Side Effects?	
				Yes	No
Nicotine Patch	○	○		○	○
Nicotine Gum	○	○		○	○
Nicotine Lozenge	○	○		○	○
Nicotine Inhaler	○	○		○	○
Zyban (Wellbutrin, Bupropion)	○	○		○	○
Chantix (Varenicline)	○	○		○	○

Have you used any of these medications in the past 30 days? ○ **Yes** ○ **No**

Review of Systems

General:

- ○ None of these apply
- ○ Unexplained weight loss or gain
- ○ Fever or chills
- ○ Fatigue / lack of energy
- ○ Weakness
- ○ Trouble sleeping

Skin:

- ○ None of these apply
- ○ Rashes
- ○ Lumps
- ○ Color change
- ○ Hair and nail changes

Head:

- ○ None of these apply
- ○ Headache
- ○ Head injury

Ears:

- ○ None of these apply
- ○ Decreased hearing
- ○ Earache
- ○ Ringing in the ears

Eyes:

- ○ None of these apply
- ○ Vision problems
- ○ Specks
- ○ Blurry or double vision
- ○ Flashing lights
- ○ Redness
- ○ Pain

Nose:

- ○ None of these apply
- ○ Stuffiness
- ○ Discharge
- ○ Itching
- ○ Sinus pain
- ○ Nose bleeds

Throat:

- ○ None of these apply
- ○ Teeth/gum problems
- ○ Dentures
- ○ Hoarseness
- ○ Sore tongue
- ○ Dry mouth
- ○ Sore throat
- ○ Thrush
- ○ Non-healing sores
- ○ Difficulty swallowing

Neck:

- ○ None of these apply
- ○ Lumps
- ○ Stiffness
- ○ Pain
- ○ Swollen glands

Respiratory:

- ○ None of these apply
- ○ Cough (dry or wet, productive)
- ○ Shortness of breath
- ○ Coughing up blood
- ○ Painful breathing
- ○ Wheezing

Cardiovascular:

- ○ None of these apply
- ○ Chest pain or discomfort
- ○ Tightness
- ○ Heart pounding/fluttering/ palpitations
- ○ Difficulty breathing lying down
- ○ Swelling
- ○ Shortness of breath with activity
- ○ Suddenly awaking from sleep with shortness of breath

Gastrointestinal:

- ○ None of these apply
- ○ Swallowing difficulties
- ○ Heartburn
- ○ Constipation
- ○ Vomiting
- ○ Change in bowel habits
- ○ Rectal bleeding
- ○ Diarrhea
- ○ Stomach pain
- ○ Yellow eyes or skin
- ○ Change in appetite
- ○ Nausea

Urinary:

- ○ None of these apply
- ○ Frequency
- ○ Urgency
- ○ Blood in urine
- ○ Pain with urination
- ○ Change in urinary strength
- ○ Incontinence

Vascular:

- ○ None of these apply
- ○ Calf pain with walking
- ○ Leg cramping
- ○ Leg pains

Musculoskeletal:

- ○ None of these apply
- ○ Muscle or joint pain
- ○ Stiffness
- ○ Back pain
- ○ Swelling of joints
- ○ Trauma

Neurologic:

- ○ None of these apply
- ○ Dizziness
- ○ Fainting
- ○ Tingling
- ○ Weakness
- ○ Numbness
- ○ Tremor
- ○ Shaking episodes

Hematologic:

- ○ None of these apply
- ○ Bruise easily
- ○ Bleed easily

Endocrine:

- ○ None of these apply
- ○ Heat or cold intolerance
- ○ Sweating
- ○ Frequent urination
- ○ Thirst
- ○ Change in appetite

Psychiatric:

- ○ None of these apply
- ○ Nervousness
- ○ Memory loss
- ○ Feeling down

Females only

- ○ None of these apply
- ○ Pregnant or currently breast feeding

Appendix 2: Smoking History

Please answer the following questions are about your history of smoking, use of other products and medications.

1. How many cigarettes have you smoked per day, on average? _____ [1-99]

2. How many years have you smoked? ________ [1-99]

3. Do you usually smoke menthol or non-menthol cigarettes?

    1 = Non-Menthol
    2 = Menthol
    3 = Both
    0 = Don't know

4. Which of the following tobacco products have you used in the last week? (Check all that apply)

    1 = Cigarettes
    2 = Cigars
    3 = Pipes
    4 = Hookah
    5 = Cigarillos
    6 = E-cigarettes
    7 = Chewing tobacco
    8 = Snuff
    9 = Other

5. During the last week did you use any of the following drugs? (Check all that apply)

    1 = Marijuana
    2 = Cocaine/Crack
    3 = Meth
    4 = PCP
    5 = Heroin
    6 = Opiods such as Morphine, Oxycotin, Percocet, Vicodini, Fentanyl
    7 = Benzodiazepines such as Valium, Xanax, Klonopin
    8 = Other

Appendix 3: Financial Questions

These next 10 questions are about your finances, medical coverage, and benefits.

1. What is your Gross Yearly Income (include all forms of income)?
   1= Less than $10,000
   2= $10,000 – $24,999
   3= $25,000 – $49,999
   4= $50,000 – $74,999
   5= $75,000 – $99,999
   6= Over $100,000
   0 = Don't know

2. What is your means of paying for medical services? (Check all that apply)
   1 = Private Insurance: HMO, PPO, CMO
   2 = Medicare
   3 = Medicaid
   4 = Veteran's Benefit
   5 =No insurance
   6 = Other
   7 = Don't know
   8= Not retired

3. Which one of the following statements best describes your personal financial situation?
   1 = I am in good shape. I am able to save and plan for the future.
   2 = I am okay. I am saving a little and I am able to provide for my needs.
   3 = I am barely getting by. I have to budget carefully and am not able to plan for the future.
   4 = I am falling behind. I have to use savings or go further into debt to pay my bills.
   5 = I am in serious financial trouble and can't quite see how I am going to make it.
   0 = Don't know

4. What is the highest grade or year of school you completed?
   1= Less than high school
   2= GED
   3= High School
   4= Some college, but no degree
   5= Associate's Degree (2 year degree)
   6= Bachelor's degree (4 year degree)
   7= Graduate education

Appendix 4: Cost of Smoking

These next questions are about the cost of cigarettes to you.

1. How do you usually buy your cigarettes?
   1 = By the Pack
   2 = By the Carton
   3 = Individually
   0 = Don't know

IF 1 = go to Question 43a<br>IF 2 = go to Question 43b<br>IF 3 = go to Question 43c

1a. How much did you pay for the LAST PACK of cigarettes you bought?

$___ __ . ____ ____ $1.00-$10.00)

1b. How much did you pay for the LAST CARTON of cigarettes you bought?

$___ __ . ___ ___ ($1.00 – $99.00)

1c. How much did you pay for the LAST CIGARETTE you bought?

. ___ __¢ (0-99¢)

44. Calculate: Cost of 1 cigarette: (Pack holds 20 cigs – so divide by 20) (Carton holds 200 so divide by 200) (do not report)<br><br>44a. Calculate Cigs per day x cost of each cig = Cost per day. Report Cost per day<br><br>44b. Calcualte Cost per week = cost per day x 7. Report cost per week<br><br>44c.Calcualte Cost per month = cost per day x 30. Report cost per month<br><br>44d. Calculate Cost per year = Cost per day x 365. Report cost per year.

Appendix 5: Medication Intolerance Questions

The next questions will help us determine which medications might be appropriate for you.

1. Are you willing to use the Nicotine Patch?
   1 = I am willing to use
   2 = I would consider using it
   0 = Don't know

2. Are you willing to use nicotine gum or nicotine lozenge?
   1 = I am willing to use
   2 = I would consider using it
   0 = Don't know
   IF male, skip questions 3&4.

3. Which best describes any potential plans to get pregnant?
   1 = Not applicable (post-menopausal, hysterectomy)
   2 = I am pregnant now
   3 = I hope to get pregnant in the next 12 months
   4 = I hope not to get pregnant in the next 12 months

4. Are you currently nursing?
   1 = YES
   2 = NO

5. Have you been diagnosed with any of the following conditions? (Check all that apply)
   1 = Schizophrenia
   2 = Bipolar Disorder
   3 = Closed Angle Glaucoma
   4 = Brain Cancer or Cerebral Aneurysm
   5 = Stroke
   6 = Seizure
   0 = I have none of these conditions

6. Do you currently take anti-seizure medications such as Dilantin, Keppra, Depakote, Tegretol, Lyrica, Topamax, or Lamictal?
   1 = YES
   2 = NO
   0 = Don't know

7. Do you currently take an MAO Inhibitor such as Linezolid, Isoniazid, Selegiline, Rasagiline, Isocarboxazid, Phenelzine, or Tranylcypromine?
   1 = YES
   2 = NO
   0 = Don't know

8. Are you willing to use Chantix (varenicline)?
   1 = I am willing to use
   2 = I would consider using it
   0 = Don't know

9. Are you willing to use Wellbutrin (bupropion)?
   1 = I am willing to use
   2 = I would consider using it
   0 = Don't know

Appendix 6: Health and Surgery

The next several questions are about your health.

1. Are you planning to have surgery?
    1= I am not scheduled for surgery and do not think I will need surgery in the future.
    2= I am scheduled for surgery
    3= I will probably need surgery, but it is not scheduled
    4 = Other

IF the answer to 1 = 2, ask question 2.

2. What is the date of the scheduled surgery _______________

Appendix 7: Cancer Questions

The next questions are about cancer diagnosis and treatment

1. Are you currently undergoing diagnosis, treatment, or follow up for cancer or possible cancer?
    1 = Yes
    2 = No

Appendix 8: Demographics

These next three questions are about you.

1. What is your race?
    1 = American Indian or Alaskan Native
    2 = Asian
    3 = Black or African American
    4 = Native Hawaiian or Pacific Islander
    5 = White
    6 = Other
    0 = Don’t know

2. Do you consider yourself to be Hispanic or Latino?
    1=YES
    2=NO
    0 = Don’t know

3. What is your gender?
    1= Male
    2= Female

Appendix 9: Alcohol Use Disorders Identification Test (AUDIT-C)(Bush, Kivlahan, McDonell, Fihn, & Bradley, 1998)

The next questions are about your alcohol use.

1. How often do you have a drink containing alcohol?
   - 0= Never
   - 1= monthly or less
   - 2= 2-4 times a month
   - 3= 2-3 times a week

2. How many standard drinks containing alcohol do you have on a typical day?
   - 0= 1 or 2
   - 1= 3 or 4
   - 2= 5 or 6
   - 3= 7 to 9
   - 4= 10 or more

3. How often do you have six or more drinks on one occasion?
   - 0 = Never
   - 1 = Less than monthly
   - 2 = Monthly
   - 3 = Weekly
   - 4 = Daily or almost daily

4. Calculate: Add all scores of 3 question above together.

Then reference score to gender:

3 or higher for females = likely alcohol abuse or dependence

4 or higher for males = likely alcohol abuse or dependence

Appendix 10: Drug Use Question

1. How many times in the past year have you used an illegal drug or used a prescription medication for non-medical reasons? ___________

Appendix 11: Recent Smoking

The follow questions are about your recent smoking

1. In the last 7 days, have you smoked at all - even a puff?
   1=YES
   2 = No
   0 = Don't know

2. In the last 7 days, how many cigarettes have you smoked per **day?**

_____ [0-99]

3. In the last 30 days, have you smoked at all- even a puff?
   1= YES
   2 = No

4. On average, how many cigarettes have you smoked per day over the last 30 days?
____

Adherence questions- These questions will be used for phone-based assessments. Medication adherence for in-person visits will be assessed via diaries, which is found in Appendix 20.

(CTA Dialogue)

Medications that have been given to you for this study should be taken as follows

- Patch: put on in AM and wear for 24 hours unless otherwise directed
- Study pills: are each taken two times per day, in the morning and in the evening unless otherwise directed by study personnel

1. Did you choose to use the nicotine patch or varenicline?

   1= Nicotine Patch

   2= Varenicline

If "Nicotine Patch" selected ,go to questions 1a-1c.

If “Varenicline” selected, go to questions 2a-2c

1a) Over the last seven days, how many days have you used the nicotine patch as prescribed?

1b) Over the last seven days, how many days have you used the pills as prescribed?

1c) If answer to 1a or 1 b is less than 7 (100% adherence), please provide reason why you did not follow the directions above.

2a) Over the last seven days, how many days have you taken the varenicline pills as prescribed above?

2b) Over the last seven days, how many days have you taken the bupropion pills as prescribed above?

2c) If answer to 2a or 2b is less than 7 (100% adherence), please provide reason why you did not follow the directions above.

Appendix 12: Work Questions

The next few questions are about work.

1. Please describe your employment status. Are you currently…?

=

1 = Working full-time (21-40 hours per week)
2 = Working Part-time (20 hours per week or less)
3 = Unemployed and looking for work
4. Disability or medical leave
5 = Retired
6 = Other
7 = Don't know

2. If you work,how many days of work have you missed due to illness in the past 30 days?

_______Days [1-30]

3. If you work, how many smoke breaks do you take during a typical workday?

______Breaks (0-9)

4. How long, on average, does each smoke breaks last? ________ (0-60 minutes)

Appendix 13: Predictor Questions

1. On a scale from 1 to 7 where 1 is “not at all confident” and 7 is “extremely confident,” how confident are you that you can quit?
16

Not at all confident						Extremely confident
1	2	3	4	5	6	7

2. On a scale from 1 to 7 where 1 is “not at all” and 7 is “a lot,” how much do you want to quit?

Not at all						A lot
1	2	3	4	5	6	7

3. Calculate: Add scores of questions above together.

2-5 = Low Efficacy

6-10 = Moderate Efficacy

11-14 = High Efficacy

Appendix 14: Fagerström Test of Nicotine Dependence (FTND)(Heatherton, Kozlowski, Frecker, & Fagerstrom, 1991)

1. How soon after you wake up do you smoke your first cigarette?
   3= Within 5 minutes
   2= 6-30 minutes
   1=31-60 minutes
   0= More than 60 min

2. Do you find it difficult to refrain from smoking in places where it is forbidden, (e.g. in church, at the library, in the cinema)?
   0 = No
   1 = Yes

3. Which cigarette would hate most to give up?
   1 = The first one in the morning
   0 = All others

4. How many cigarettes per day do you smoke?
   0= 10 or less
   1= 11-20
   2= 21-30
   3= 31 or more

5. Do you smoke more frequently during the first hours after waking than the rest of the day?
   0 = No
   1 = Yes

6. Do you smoke when you are so ill that you are in bed most of the day?
   0 = No
   1 = Yes

Calculate: Add scores of all 6 items.

1-2 = low dependence
3-4 = low-moderate dependence
5-7 = moderate dependence
8+ = high dependence

Appendix 15: Mood and Physical Symptoms Scale-2 (MPSS-2)(West & Hajek, 2004)

The next questions are about how you have been feeling over the past 24 hours.

1. How much of the time have you felt the urge to smoke in the past 24 hours?
   0=Not at all
   1=A little of the time
   2=A lot of the time
   3=Almost all of the time
   4= All of the time

2. How strong have the urges been?
   0=No urge
   1=Slight
   2=Moderate
   3=Strong
   4=Very Strong
   5=Extremely Strong

3. Calculate: Please add scores of questions above<br><br>0-2 = Mild<br>3-6 = Moderate<br>7-10 = Severe

Appendix 16: Patient Health Questionnaire-2 (PHQ-2)(K. Kroenke, Spitzer, & Williams, 2003) and question 9 of Patient Health Questionnaire-9 (K. Kroenke et al., 2003; Kroenke, Spitzer, & Williams, 2001)

Over the past two weeks, how often have you been bothered by any of the following problems?

	Not at all	Several days	More than half the days	Nearly every day
1. Little interest or pleasure in doing things	0	1	2	3
2. Feeling down, depressed or hopeless	0	1	2	3
9. Thoughts that you would be better off dead or hurting yourself in some way				

Scoring

If the sum of the answers to 1 and 2 are ≥ 3, a mood disorder is indicated.

If the sum of the answers to 1 and 2 are < 3, a mood disorder is not indicated.

Appendix 17: Generalized Anxiety Disorder 2-item Scale (GAD-2) (Plummer, Manea, Trepel, & McMillan, 2016)

**GAD-7**<br>**Over the <u>last 2 weeks</u>, how often have you been bothered by any of the following problems?**	Not at all	Several days	More than half the days	Nearly every day
1. Feeling nervous, anxious or on edge	0	1	2	3
2. Not being able to stop or control worrying	0	1	2	3

.

.

3. Calculate: Add scores of GAD-7 Responses together for total
0-2= Negative screen
3-6= Positive screen

Appendix 18: Perceived Stress Scale (PSS-4)(Cohen, Kamarck, & Mermelstein, 1983)

The next set of questions asks you about your feelings and thoughts during the last month. In each case, please indicate with a check how often you felt or thought a certain way.

1. In the last month, how often have you felt that you were unable to control the important things in your life?
   0=never
   1=almost never
   2=sometimes
   3=fairly often
   4=very often

2. In the last month, how often have you felt confident about your ability to handle your personal problems?
   0=never
   1=almost never
   2=sometimes
   3=fairly often
   4=very often

3. In the last month, how often have you felt that things were going your way?
   0=never
   1=almost never
   2=sometimes
   3=fairly often
   4=very often

4. In the last month, how often have you felt difficulties were piling up so high that you could not overcome them?
   0=never
   1=almost never
   2=sometimes
   3=fairly often
   4=very often

5. Calculate: Add scores of above responses

0-5 = Low Stress
6-11 = Moderate Stress
12-16 = High Stress

Appendix 19: Phone-Based Assessments

Abstinence Status

1. Have you smoked any cigarettes in the last 24 hours? (Call 4)
2. How many cigarettes have you smoked in the last 24 hours? (Call 4)
3. Have you smoked cigarettes in the last 7 days? (Call 1,2,3, 4, 5, 6, 7, 8, 9, 10)
4. On average, how many cigarettes have you smoked per day in the 7 days week? (Call 1,2,3, 4, 5, 6, 7, 8, 9, 10)
5. Have you smoked cigarettes in the last 30 days? (Calls 7, 8, 9, 10)
6. On average, how many cigarettes have you smoked per day over the last 30 days? (Calls 7, 8, 9, 10)

Medications and Side Effects

1. Are you experiencing any side effects from the medications? Yes No
2. If yes, what side effect(s) are you experiencing?

Abnormal Dreams	Dizziness/Lightheadedness	Anxiety
Constipation	Headache	Agitation/Anger
Rash	Insomnia	Vomiting
Dry Mouth	Nausea	Gas
	Seizures	Other (Please describe)

3. How severe?

1. Mild	6.
2.	7.
3.	8.
4.	9.
5.	10. Severe

4. When did it start?
5. How long did it last (in days)?
6. Did you stop medication?

If **more than one side effect** reported, repeat questions 3-6.

Withdrawal and Urge Questions

1. On a scale from 1 to 10, how strong have your urges to smoke been over the last week?

1. Not at all strong	6.
2.	7.
3. A little strong	8. Very strong
4.	9.
5. Moderately strong	10. Extremely strong

2. On a scale from 1-10, how bad have your withdrawal symptoms (for example, irritability or agitation that occur when you don't smoke) been over the last week?

1. Not at all stressed
2.
3. A little stressed
4.
5. Moderately stressed
6.
7.
8. Very stressed
9.
10. Extremely stressed

Confidence in Ability and Motivation to Become Tobacco-Free

1. On a scale of 1-10, how motivated are you to quit smoking or stay quit if abstinent?

1. Not at all motivated
2.
3. A little motivated
4.
5. Moderately motivated
6.
7.
8. Very motivated
9.
10. Extremely motivated

2. On a scale of 1-10, how confident are you that you can quit if abstinent or stay quit if abstinent?

1. Not at all confident
2.
3. A little confident
4.
5. Moderately confident
6.
7.
8. Very confident
9.
10. Extremely confident

26- and 52-Week Call Questions

1. Since completing your last visit with us, have you used any smoking cessation medications? (Nicotine patches, gum/lozenge, inhaler, Chantix, Wellbutrin, bupropion, or Zyban) Yes No

2. Since completing your last visit with us, have you smoked at all, even a puff? Yes No

Appendix 20: 7 Day Smoking History and Medication Adherence Diary

Please record your medication use and smoking each day, starting seven days before your next visit. (Will be adjusted based on medication regimen chosen and specific in-person visit)

	Date	# cigs smoked?	Did you take your **Chantix** pills today?		Did you take your **Bupropion/placebo** pills today?	
			First Dose	Second Dose	First Dose	Second Dose
**1**	_____/_____	_______	Pill 1: ○ YES ○ NO	Pill 2: ○ YES ○ NO	Pill 1: ○ YES ○ NO	Pill 2: ○ YES ○ NO
**2**	_____/_____	_______	Pill 1: ○ YES ○ NO	Pill 2: ○ YES ○ NO	Pill 1: ○ YES ○ NO	Pill 2: ○ YES ○ NO
**3**	_____/_____	_______	Pill 1: ○ YES ○ NO	Pill 2: ○ YES ○ NO	Pill 1: ○ YES ○ NO	Pill 2: ○ YES ○ NO
**4**	_____/_____	_______	Pill 1: ○ YES ○ NO	Pill 2: ○ YES ○ NO	Pill 1: ○ YES ○ NO	Pill 2: ○ YES ○ NO
**5**	_____/_____	_______	Pill 1: ○ YES ○ NO	Pill 2: ○ YES ○ NO	Pill 1: ○ YES ○ NO	Pill 2: ○ YES ○ NO
**6**	_____/_____	_______	Pill 1: ○ YES ○ NO	Pill 2: ○ YES ○ NO	Pill 1: ○ YES ○ NO	Pill 2: ○ YES ○ NO
**7**	_____/_____	_______	Pill 1: ○ YES ○ NO	Pill 2: ○ YES ○ NO	Pill 1: ○ YES ○ NO	Pill 2: ○ YES ○ NO
**Next Visit**	_____/_____					
