# Supplementary material for: Adaptive Smoking Cessation Using Precessation Varenicline or Nicotine Patch: A Randomized Clinical Trial
Source: JAMA Netw Open. 2023 Sep 8;6(9):e2332214. doi: 10.1001/jamanetworkopen.2023.32214 (PMC10492187; doi:10.1001/jamanetworkopen.2023.32214)
Supplement: Supplement 2. — Data Sharing Statement [file jamanetwopen-e2332214-s002.pdf]

Data Sharing Statement

Davis. Adaptive Smoking Cessation Using Precessation Varenicline or Nicotine Patch. *JAMA Netw Open*. Published September 08, 2023. doi:10.1001/jamanetworkopen.2023.32214

Data
Data available: Yes
Data types: Deidentified participant data
How to access data: De identified participant data will be made available upon reasonable request. Please contact corresponding author at james.m.davis@duke.edu
When available: With publication

Supporting Documents
Document types: None

Additional Information
Who can access the data: investigators
Types of analyses: meta analysis
Mechanisms of data availability: with investigator support
Any additional restrictions: NA
